# The GYF domain protein PSIG1 dampens the induction of cell death during plant-pathogen interactions

**DOI:** 10.1371/journal.pgen.1007037

**Published:** 2017-10-26

**Authors:** Hidenori Matsui, Yuko Nomura, Mayumi Egusa, Takahiro Hamada, Gang-Su Hyon, Hironori Kaminaka, Yuichiro Watanabe, Takashi Ueda, Marco Trujillo, Ken Shirasu, Hirofumi Nakagami

**Affiliations:** 1 RIKEN Center for Sustainable Resource Science, Yokohama, Japan; 2 Graduate School of Environmental and Life Science, Okayama University, Okayama, Japan; 3 Faculty of Agriculture, Tottori University, Tottori, Japan; 4 Department of Life Sciences, Graduate School of Arts and Sciences, The University of Tokyo, Tokyo, Japan; 5 National Institute for Basic Biology, Okazaki, Japan; 6 Department of Basic Biology, SOKENDAI (Graduate University for Advanced Studies), Okazaki, Japan; 7 Japan Science and Technology Agency (JST), PRESTO, Kawaguchi, Japan; 8 Leibniz Institute of Plant Biochemistry, Halle (Saale), Germany; 9 Max Planck Institute for Plant Breeding Research, Cologne, Germany; USDA/UC Berkeley, UNITED STATES

## Abstract

The induction of rapid cell death is an effective strategy for plants to restrict biotrophic and hemi-biotrophic pathogens at the infection site. However, activation of cell death comes at a high cost, as dead cells will no longer be available for defense responses nor general metabolic processes. In addition, necrotrophic pathogens that thrive on dead tissue, take advantage of cell death-triggering mechanisms. Mechanisms by which plants solve this conundrum remain described. Here, we identify *PLANT SMY2-TYPE ILE-GYF DOMAIN-CONTAINING PROTEIN 1 (PSIG1)* and show that *PSIG1* helps to restrict cell death induction during pathogen infection. Inactivation of PSIG1 does not result in spontaneous lesions, and enhanced cell death in *psig1* mutants is independent of salicylic acid (SA) biosynthesis or reactive oxygen species (ROS) production. Moreover, PSIG1 interacts with SMG7, which plays a role in nonsense-mediated RNA decay (NMD), and the *smg7-4* mutant allele mimics the cell death phenotype of the *psig1* mutants. Intriguingly, the *psig1* mutants display enhanced susceptibility to the hemi-biotrophic bacterial pathogen. These findings point to the existence and importance of the SA- and ROS-independent cell death constraining mechanism as a part of the plant immune system.

## Author summary

Programmed cell death (PCD) has crucial roles in development and immunity in multicellular organisms. In plants, rapid PCD induction, so-called hypersensitive response (HR) cell death, can be triggered as a part of immune system, and plays an important role in restricting pathogen growth. Despite its importance, cell death induction can backfire on plants because of the diversified infection strategies of plant pathogens. It is therefore assumed that plants have mechanisms by which they are able to minimize PCD induction during plant-pathogen interactions. However, their existence and biological significance are not clear yet. Here, we demonstrate that PSIG1, which has the GYF domain that is highly conserved among diverse eukaryotic species, restricts cell death induction during pathogen invasions. Importantly, *psig1* mutants do not display autoimmune phenotypes, and are more susceptible to the virulent bacterial pathogen. Our findings suggest that the restriction of cell death can have benefits for plants to defend themselves against hemi-biotrophic bacterial pathogen infections. We further provide evidence suggesting a mechanism by which PSIG1 may contain cell death by regulating the RNA metabolism machinery.

## Introduction

Programmed cell death (PCD) has crucial roles in development and immunity in multicellular organisms [1]. In plants, hypersensitive response (HR) cell death is one of most studied forms of PCD. The HR is a part of effector-triggered immunity (ETI), the second layer of the plant immune system, and plays an important role in restricting pathogen growth. ETI is primarily effective against biotrophic and hemi-biotrophic pathogens [2], which obtain nutrients from live host cells and actively suppress the first layer of the plant immune system, pathogen-associated molecular pattern (PAMP)-triggered immunity (PTI). By contrast, ETI-associated HR cell death may benefit necrotrophic pathogens, which often secret toxic compounds to kill host cells and obtain nutrients from dead cells [3]. Indeed, some necrotrophic pathogens promote virulence by hijacking the plant’s HR machinery [3]. The contribution of HR cell death to resistance against hemi-biotrophic pathogens, which switch from a biotrophic phase to a necrotrophic one [4], is still under debate [5–7]. Under this premise, minimizing the induction of cell death, as part of a defense response, would result in an advantage for plants against pathogens that can benefit from dead cells.

Identification of so called ‘lesion mimic mutants (LMMs)’ that display spontaneous HR-like cell death, has greatly advanced our understanding of HR cell death regulation [8]. The phytohormone SA promotes HR cell death induction, and LMM phenotypes are often compromised in SA-deficient mutants background such as the *sid2* mutant, which are not able to accumulate SA upon immune activation [9–12]. Several LMMs initiate lesion formation under specific growth conditions and/or upon chemical treatments [1]. Lesion formation of the *lsd1* mutant can be triggered by shifting plants from short day conditions to long day conditions [13]. Upon pathogen inoculation, the *lsd1* mutant displays runaway cell death (RCD) phenotype that forms lesions beyond the inoculation site [14]. *LSD1* encodes a zinc finger protein, and negatively regulate initiation of PCD and RCD, partly via maintenance of ROS homeostasis [11,15,16]. The basic region leucine zipper (bZIP) transcription factor, bZIP10, and the type I metacaspase, MC1, interact with LSD1 and regulate the PCD [6,17]. HR cell death can be regulated both positively and negatively by ROS [18–21]. Likewise, autophagy can act as both a positive and negative regulator of HR cell death, which was proposed to be dependent on plant age [22–26]. Infection of the avirulent bacterial strain, *Pseudomonas syringae* pv. *tomato* DC3000 (*Pto*) carrying *AvrRPM1* or *AvrRPS4*, triggers the autophagic activity [23]. Application of an SA agonist, benzo(1,2,3)thiadiazole-7-carbothioic acid (BTH), induces autophagosome formation [26]. The *atg5* mutant displays an RCD phenotype that depends on SA accumulation and signaling [26]. Similarly, the autophagic component *BECLIN1* is required to prevent RCD [27,28], suggesting that autophagy negatively regulates RCD. In contrast, the autophagic components positively regulates HR cell death induction upon *Pto AvrRPS4* or the avirulent oomycete *Hyaloperonospora arabidopsidis* (*Hpa*) infection [23].

PAMP receptors also play roles in HR-like cell death regulation. BRASSINOSTEROID (BR) INSENSITIVE 1-ASSOCIATED RECEPTOR KINASE 1 (BAK1), which is a co-receptor of PAMP and BR receptors, negatively regulates cell death together with its close homologue SOMATIC EMBRYOGENESIS RECEPTOR KINASE 4 (SERK4) [29–32]. The *bak1-3* and *bak1-4* mutant, null alleles of *BAK1*, are impaired in PAMP- and BR-signaling, and the *bak1-4 serk4* double mutant displays spontaneous cell death [30]. *N*-glycosylation and components of endoplasmic reticulum (ER) quality control contribute to activate the *bak1-4 serk4-*depdendent cell death [33]. The *bak1-3* and *bak1-4* alleles display RCD phenotype upon hemi-biotrophic bacterial pathogen *Pto* or necrotrophic fungal pathogen *Alternaria brassiciola* infection [32]. By contrast, the *bak1-5* allele is only impaired in PAMP-signaling, but not in BR-signaling or the SERK4-dependent cell death regulation [34]. The cell death induction in the *bak1-5* allele upon pathogen inoculation has not been characterized yet.

A mechanistic link between PCD and immune system is also suggested by the observation that the activation of PTI suppresses Fumonisin B1 (FB1)-triggered PCD. Fumonisin B1, is a mycotoxin produced by the necrotrophic fungal pathogen *Fusarium moniliforme* that induces host PCD and promotes fungal virulence [35]. This suggesting the existence of a pathway capable of restricting cell death induction [35]. Recently, a signaling sector that mediates ETI and is inhibited by PTI, namely ETI-mediating and PTI-inhibited sector (EMPIS), has been identified [36]. However, the molecular mechanisms underlying the restriction of cell death remain obscure.

The glycine-tyrosine-phenylalanine (GYF) motif represents the conserved signature of the GYF domain, which was first identified in human CD2-binding protein 2 (CD2BP2), where it was required for binding to the cytoplasmic tail of CD2 [37]. CD2BP2 plays a role in CD2-triggered T lymphocyte activation and spliceosomal protein functions [37,38]. The GYF domain is highly conserved across eukaryotic species and can be roughly classified into two subfamilies, the CD2BP2-type GYF domain and the suppressor of myosin 2 (SMY2)-type GYF domain [38,39]. Structures of the GYF domains have been determined [39,40], and the recognition motifs have been characterized in detail [41]. The GYF domain of PSIG1 (At5g42950) recognizes proline-rich sequences as do other GYF domains found in human and yeast proteins [41]. Recently, *PSIG1* was reported to be indispensable for plantago asiatica mosaic virus infection and designated as *Essential for poteXvirus Accumulation 1* (*EXA1*) [42]. Furthermore, *MUTANT*, *SNC1-ENHANCING 11* (*MUSE11*) was found to be *EXA1/PSIG1* [43]. *EXA1/MUSE11/PSIG1* was proposed to regulate levels of plant immune receptors via translational repression, and thereby negatively regulate plant immunity [43]. Nevertheless, significance of the GYF domain for functions of PSIG1 and other plant GYF domain proteins has not been addressed yet.

Here, we identify PSIG1, a plant-specific protein with the GYF domain, as a key player in the restriction of pathogen-induced PCD. Analyses of *psig1* mutants suggested that the PCD-restriction system is crucial for resistance against hemi-biotrophic bacterial pathogens.

## Results

### PSIG1 is an early PAMP-responsive phosphoprotein and negatively regulates PAMP responses in an SA-independent manner

**Fig 1:**
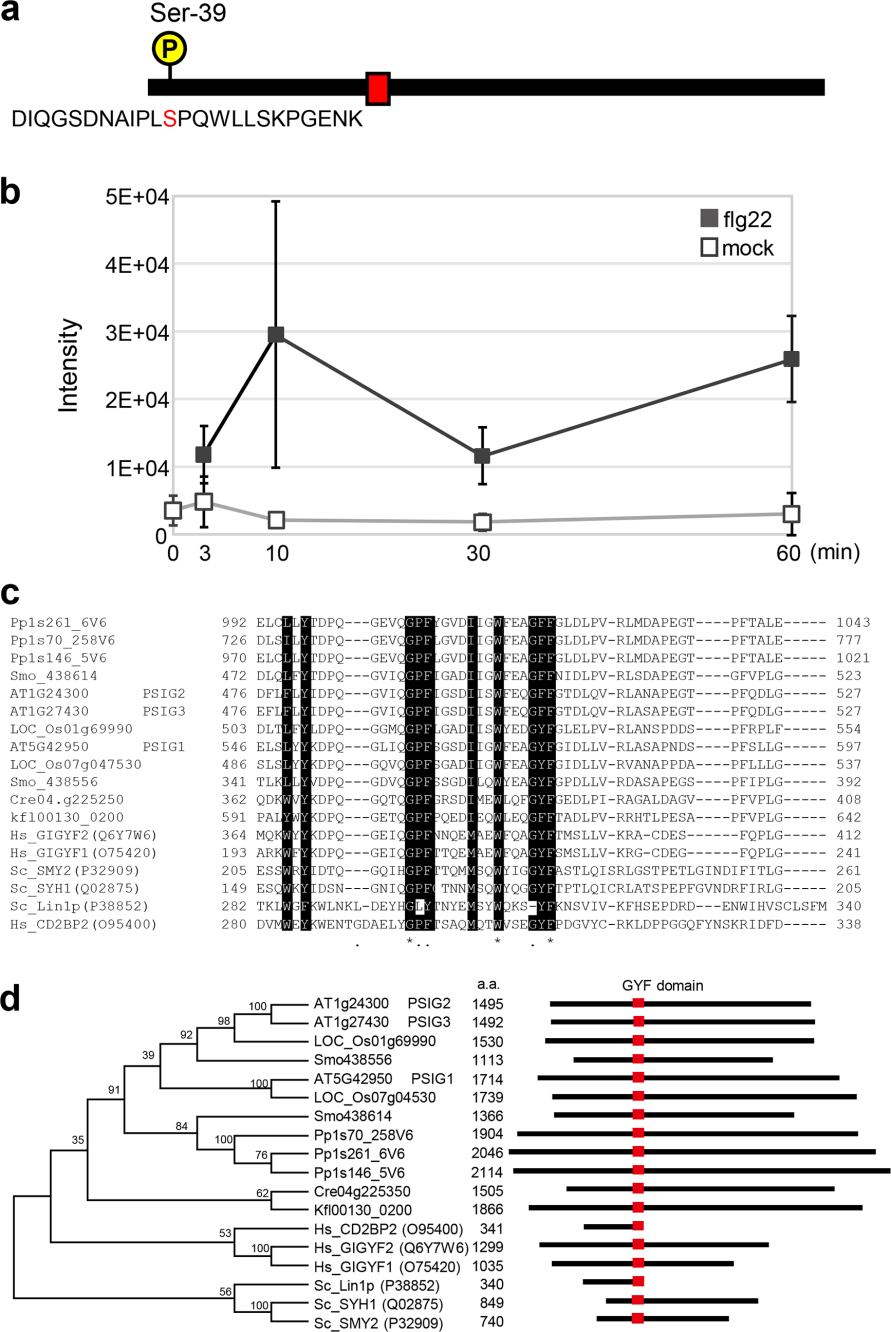
GYF domain proteins. **a**, Schematic structure and the phosphorylation site of PSIG1. Ser-39 was found to be the phosphorylation site. The red box indicates the GYF domain. **b**, Relative abundance of the ‘DIQGSDNAIPLpSPQWLLSKPGENK’ phosphopeptide upon flg22 treatment. Arabidopsis seedlings were treated with 1 μM flg22 or received a mock treatment (dH_2_O) prior to phosphoproteome analysis. Data are shown as the mean ± SD from three independent experiments. **c**, Aligned amino acid sequences of the GYF domains from diverse eukaryotic species. Key residues for the GYF domain are delineated as white text on a black background. At, Os, Smo, Phpat, Cre, Kfl, Hs and Sc stand for following species: *Arabidopsis thaliana*, *Oryza sativa*, *Selaginella moellendorffii*, *Physcomitrella patens*, *Chlamydomonas reinhardtii*, *Klebsormidium flaccidum*, *Homo sapiens* and *Saccharomyces cerevisiae*, respectively. **d**, Phylogenetic tree and schematic structures of GYF-domain proteins from diverse eukaryotes. Species abbreviations are defined in Fig 1C. Numbers on the phylogenetic tree indicate the bootstrap values. Red boxes indicate the GYF domain.

To explore for novel components in PTI, we performed a differential phosphoproteome analysis and found that the phosphorylation status of the N-terminal region of PSIG1 (At5g42950) was modulated within 10 minutes after treatment of Arabidopsis seedlings with PAMP flg22, the conserved immunogenic epitope of bacterial flagellin (Fig 1A and 1B, S19 Fig and S4 Table). PSIG1 contains the GYF domain that is highly conserved among a variety of proteins from diverse eukaryotic species (Fig 1C and S1A Fig). However, the predicted PSIG1 sequence was found to be plant specific (Fig 1D and S1B Fig).

**Fig 2:**
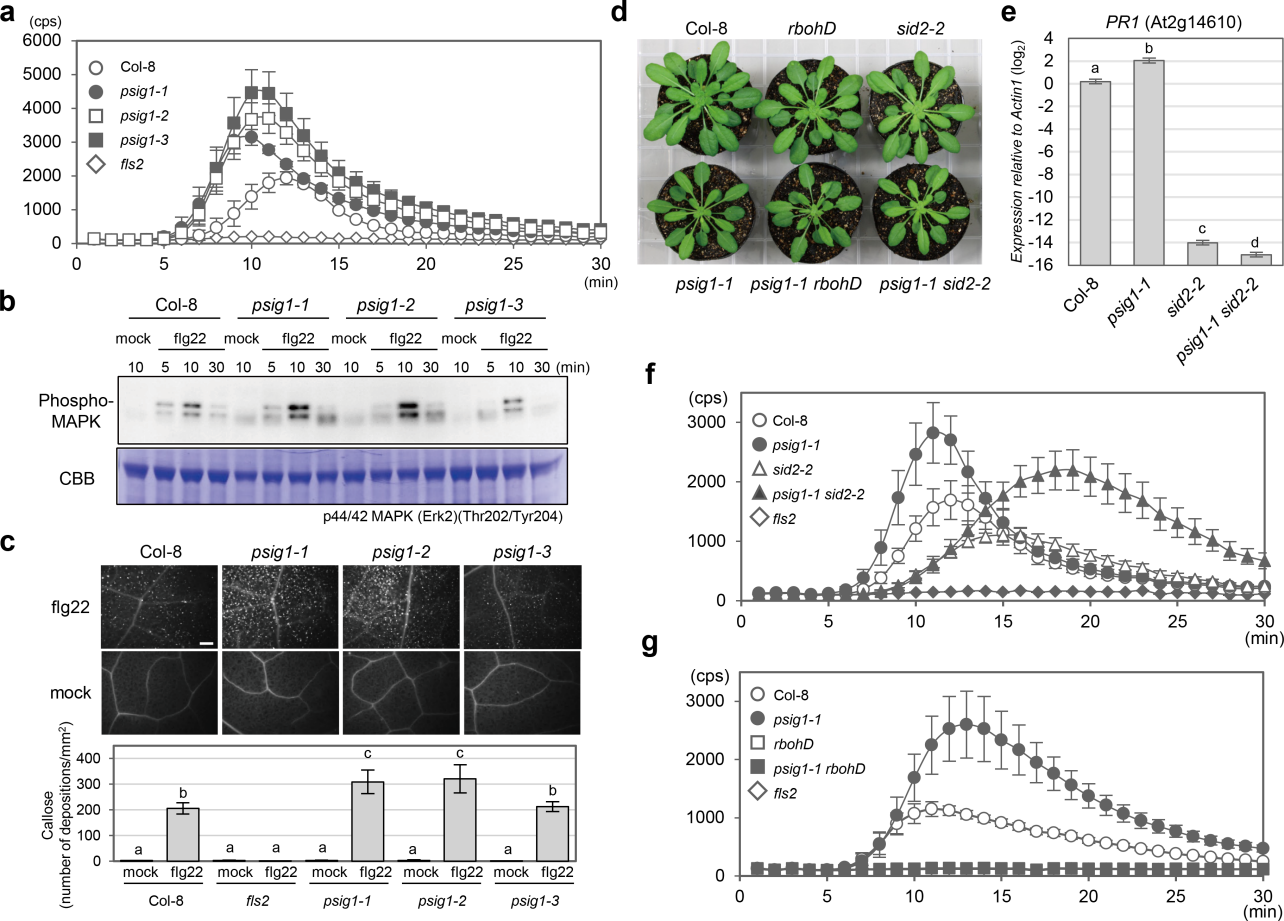
PTI responses in the *psig1* mutants. **a**, Flg22-induced ROS production in the *psig1* mutants. Data are shown as the mean ± SE. **b**, Flg22-induced MAPK activation in the *psig1* mutants. **c**, Flg22-induced callose deposition in the *psig1* mutants. Callose deposition was quantified with Image J software. Data are shown as the mean ± SE. Statistical groups were determined using the Tukey HSD test. Statistically significant differences are indicated by different letters (*p* < 0.05). The scale bar represents 200 μm. **d**, The *psig1-1* mutant has a slight dwarf phenotype. Photograph of 6-week-old plants grown under short day conditions. **e**, *PR1* gene expression in 10-day-old seedlings. Data are shown as the mean ± SE. Statistical groups were determined using the Tukey HSD test. Statistically significant differences are indicated by different letters (*p* < 0.01). **f**, Flg22-induced ROS production in the *psig1-1 sid2-2* mutants. Data are shown as the mean ± SE. **g**, Flg22-induced ROS production in the *psig1-1 rbohD* mutants. Data are shown as the mean ± SE.

The functions of plants proteins containing the GYF-domain are largely unknown. We therefore isolated three independent T-DNA insertion mutants to study *PSIG1’s* functions (S2 Fig). Treatment with flg22 enhanced oxidative bursts for all three *psig1* mutant alleles when compared to wild-type (WT) Col-8 plants (Fig 2A and S3A Fig). Similarly, mitogen-activated protein kinase activation and callose deposition were also enhanced in the *psig1-1* and *psig1-2* alleles, but were similar to WT for *psig1-3* (Fig 2B and 2C and S3B–S3D Fig). Both the *psig1-1* and *psig1-2* alleles displayed a weak dwarf phenotype mostly absent in *psig1-3* (S2A Fig). Dwarf phenotypes are often associated with the inappropriate activation of the SA pathway [44]. Indeed, expression of the SA-related marker gene *PATHOGENESIS-RELATED 1* (*PR1*) was upregulated in the *psig1* mutants under normal growth conditions (Fig 2E and S4A and S4B Fig). Therefore, to investigate the contribution of SA signaling to the phenotypes, the mutant having the strongest phenotype, *psig1-1*, was crossed with the SA biosynthesis-deficient *sid2-2* mutant [45]. Unexpectedly, the enhanced oxidative burst phenotype was not suppressed in the *psig1-1 sid2-2* double mutant. Instead, the oxidative burst was delayed (Fig 2F and S5C Fig), and in addition, the dwarf phenotype was unaffected by the *sid2-2* introgression (Fig 2D). The expected decrease in SA levels caused by *sid2-2* in the double mutant, was confirmed by analyzing *PR1* expression (Fig 2E). Phenotypes of *psig1-1* were complemented by the expression of *PSIG1* driven by its native promoter (S6A–S6C Fig), indicating that *PSIG1* negatively regulates PAMP responses and positively plant growth in an SA-independent manner.

### *PSIG1* is required for resistance against virulent hemi-biotrophic bacterial pathogen

**Fig 3:**
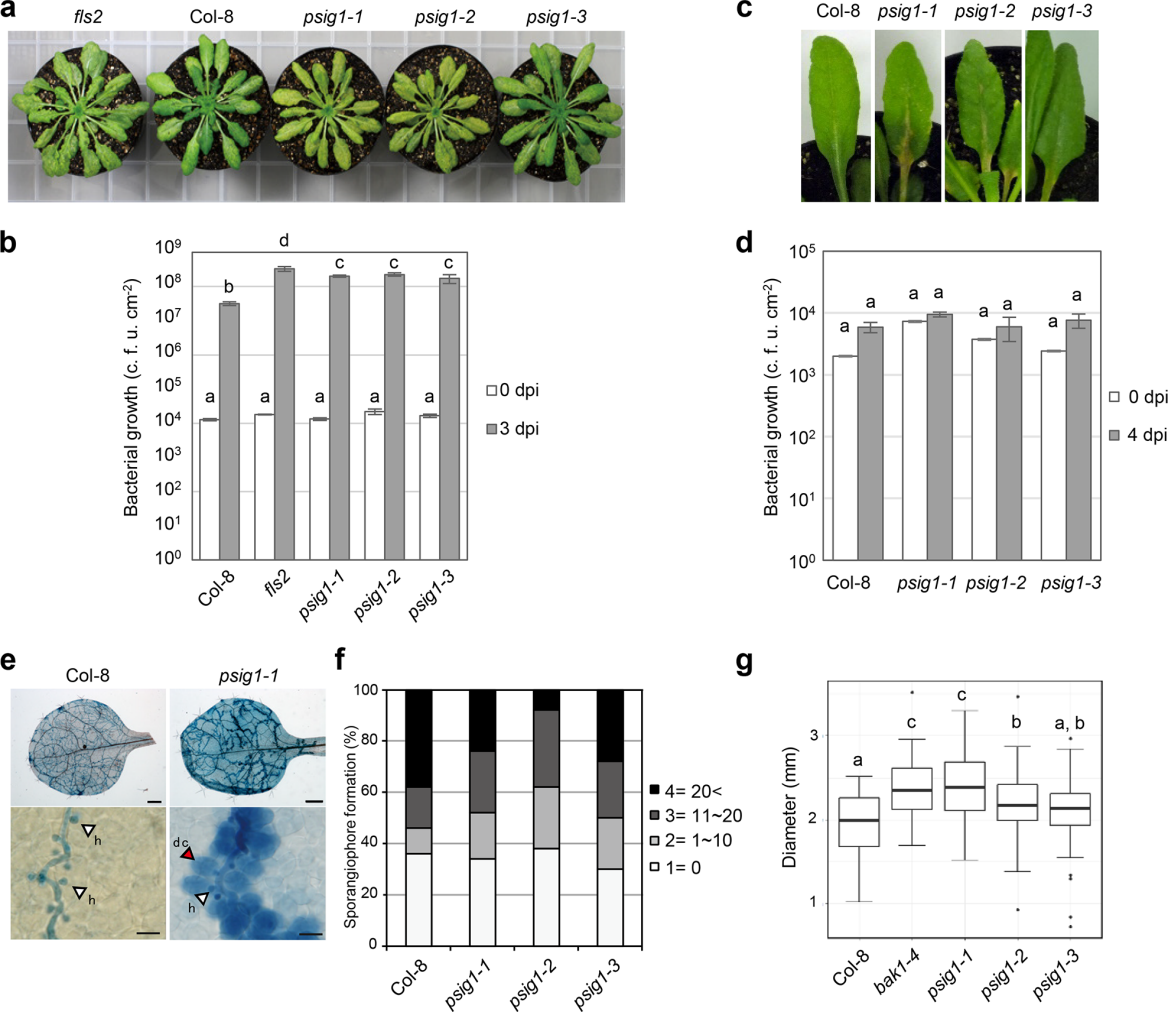
The *psig1* mutant phenotypes against pathogens. **a**, Photograph of *Pto*-infected plants. Six-week-old plants were spray inoculated with 1 x 10^8^ c.f.u. ml^-1^ of *Pto* and photographed 3 days after inoculation. **b**, The *psig1* mutants were more susceptible to *Pto*. Plants were spray inoculated with *Pto*, and bacterial growth was determined at 0 and 3 dpi. Data are shown as the mean ± SE. Statistical groups were determined using the Tukey HSD test. Statistically significant differences are indicated by different letters (*p* < 0.05). **c**, Photograph of *Pto AvrRPM1*-infected leaves. Six-week-old plants were spray inoculated with 5 x 10^8^ c.f.u. ml^-1^ of *Pto AvrRPM1* and photographed 4 days after inoculation. **d**, The *psig1* mutants did not display reduced or enhanced susceptibility against *Pto AvrRPM1*. Plants were spray inoculated with *Pto AvrRPM1*, and bacterial growth was determined at 0 and 4 dpi. Data are shown as the mean ± SE. Statistical groups were determined using the Tukey HSD test. Statistically significant differences are indicated by different letters (*p* < 0.05). **e**, Photograph of *Hpa* Noco2-infected leaves. Plants were inoculated with *Hpa* Noco2, and true leaves were stained with trypan blue 6 days after inoculation. White arrowheads indicate infection hyphae of *Hpa* Noco2 and a red arrowhead indicates dead cell. Scale bars in upper panels and lower panels indicate 1 mm and 100 μm, respectively. **f**, The *psig1* mutants were less susceptible to *Hpa* Noco2. Fourteen-day-old seedlings were inoculated with spores of Hpa Noco2, and the number of sporangiophores on true leaves was scored (0 = 1, 1–10 = 2, 11–20 = 3, >20 = 4) 6 days after inoculation. Bars show the percentage of leaves for each score (n = 25). **g**, Boxplots represent lesion size (n = 63 to 66). Boxes show upper and lower quartiles of the data, and black lines represent the medians. Statistical groups were determined using the Tukey HSD test. Statistically significant differences are indicated by different letters (*p* < 0.05).

Because the *psig1* mutants displayed enhanced responsiveness to flg22, we hypothesized that this would result in increased pathogen resistance. To our surprise, the *psig1* mutants displayed enhanced susceptibility against the virulent hemi-biotrophic bacterial pathogen *Pseudomonas syringae* pv. *tomato* DC3000 (*Pto*) (Fig 3A and 3B and S5A, S5B and S6D Figs). The enhanced susceptible phenotype may reflect stomatal defects, as plants were spray inoculated. To test this possibility, we analyzed stomata density and flg22-induced stomatal closure. However, *psig1-1* responses were comparable to that of WT plants (S7 Fig).

### Atypical induction of cell death appears in the *psig1* mutants

**Fig 4:**
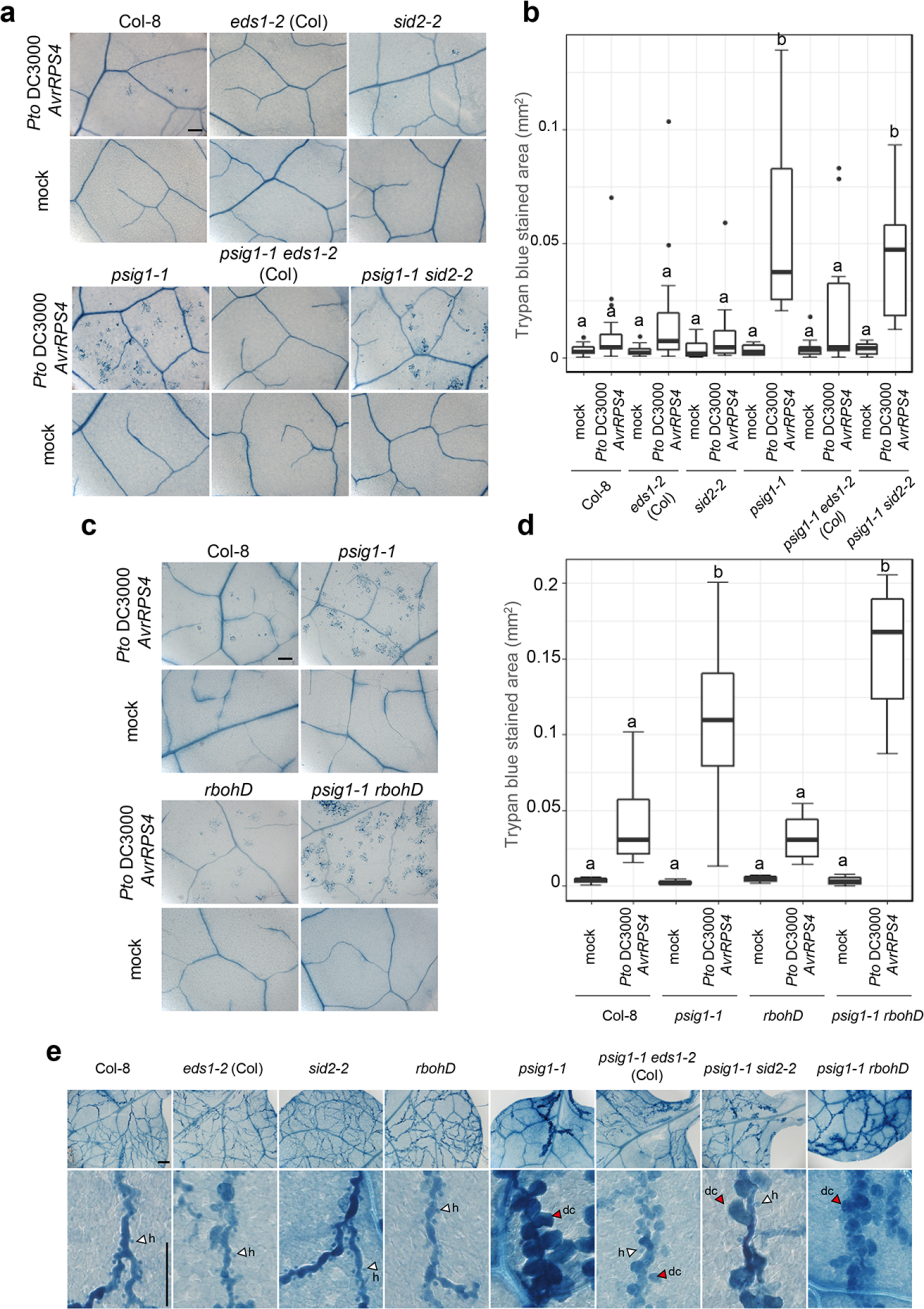
*PSIG1* negatively regulates the induction of cell death during pathogen infection. **a** and **c**, Induction of RPS4-triggered cell death was pronounced in the *psig1-1* mutant in an SA and ROS-independent manner. Plants were spray inoculated with 1 x 10^8^ c.f.u. ml^-1^ of *Pto AvrRPS4*, and dead cells were visualized by trypan blue staining 2 days after inoculation. The scale bar represents 200 μm. **b** and **d**, Trypan blue stained area. Plants were spray inoculated with 1 x 10^8^ c.f.u. ml^-1^ of *Pto AvrRPS4*, and dead cells were visualized by trypan blue staining 2 days after inoculation. The stained area was measured using an imaging software. Two to 3 leaves were taken from each of at least 5 individual plants for **b**. Three leaves were taken from each of 3 individual plants for **d**. The box plot indicates the area of trypan blue stained cells. Boxes show upper and lower quartiles of the data, and black lines represent the medians. Statistical groups were determined using the Tukey HSD test. Statistically significant differences are indicated by different letters (*p* < 0.05). **e**, The *psig1-1* mutant induces cell death upon *Hpa* Noco2 infection. Plants were inoculated with spores of *Hpa* Noco2, and dead cells on true leaves were visualized by trypan blue staining 5 days after inoculation. White arrowheads indicate infection hyphae of *Hpa* Noco2 and red arrowheads indicate dead cells. Scale bars in the upper and lower panels indicate 200 μm and 100 μm, respectively.

To investigate the role of *PSIG1* in plant immunity, we challenged the mutant plants with different types of pathogens. Bacterial growth was unaffected in the *psig1* mutants spray inoculated with the avirulent strain *Pto* carrying *AvrRPM1* (*Pto AvrRPM1*) (Fig 3D). Instead, we observed the development of visible cell death symptoms 4 days after *Pto AvrRPM1* inoculation in the *psig1* mutants, which were absent in WT plants (Fig 3C). Infected leaves were stained with trypan blue to visualize dead cells, revealing that cell death induction was significantly increased in the *psig1* mutants compared to WT plants (S8A and S8B Fig). Similar levels of cell death induction were observed in the *psig1-1* mutant after inoculation with *Pto* carrying *AvrRPS4* (*Pto AvrRPS4*) that activates effectual ETI in Col-0 without macroscopically visible HR cell death (Fig 4A–4D and S8F and S8G Fig) [46]. Cell death was not observed in the *psig1* mutants under normal growth conditions without pathogen inoculation (Fig 4A–4D and S8–S11 Figs), in difference to the typical LMMs that develop spontaneous cell death without pathogen attack [8]. It suggests that *PSIG1* function is dedicated to the restriction of cell death after activation.

The Col-8 ecotype is susceptible to obligate biotrophic oomycete *Hyaloperonospora arabidopsidis* (*Hpa*) isolate Noco2, meaning that penetrated cells generally do not trigger HR cell death (Figs 3E and 4E). However, infection with the virulent *Hpa* Noco2, resulted in the induction of cell death in the *psig1* mutants (Figs 3E and 4E and S6E Fig). In spite of the cell death, the *psig1* mutants were not fully resistant against *Hpa* Noco2 (Figs 3E, 3F and 4E and S6E and S6F Fig). This observation contrasts to incompatible interactions, where penetrated cells trigger a fast HR cell death response, thereby limiting further pathogen growth [47]. Of note, RCD was not observed in the *psig1* mutants, and cell death induction was restricted to cells in contact with the *Hpa* Noco2 hyphae, and therefore, potentially penetrated (Figs 3E and 4E). Our observations that *Hpa* Noco2 grew in spite of HR-like cell death may be reconciled by the possibility that pathogen growth was faster than the cell death. Together, these results suggest that *PSIG1* negatively regulates the induction of cell death in pathogen-targeted cells.

### *PSIG1* suppresses the induction of ETI-triggered cell death in an SA- and ROS-independent manner

We found that the frequency of cell death after inoculation with the avirulent bacteria *Pto AvrRPS4* markedly increased in the *psig1* mutants compared to WT plants (Fig 4A–4D). It was not clear, however, whether the observed cell death in the *psig1* mutants was associated with the activation of AvrRPS4-dependent ETI. AvrRPS4 is recognized by the TIR-NB-LRR-type receptor pair RRS1/RPS4 [48], and EDS1 is required for RRS1/RPS4-triggered ETI [49]. Therefore, we generated *psig1-1 eds1-2* double mutant and inoculated it with *Pto AvrRPS4*. As expected, *AvrRPS4*-triggered cell death was suppressed in the *psig1-1 eds1-2* mutant (Fig 4A and 4B). In addition, *AvrRPM1*-triggered cell death, which does not require EDS1, was unaffected in the *psig1-1 eds1-2* mutant (S9 Fig). These results indicate that the induction of ETI-triggered cell death is increased in the *psig1* mutants.

Since SA potentiates the HR [9], increased SA levels in the *psig1* mutants could result in the enhanced induction of ETI-triggered cell death. To examine this possibility, the *psig1-1 sid2-2* mutant was challenged with *Pto AvrRPS4* or *Pto AvrRPM1*. The enhanced cell death phenotype of *psig1-1* was retained in the *sid2-2* background (Fig 4A and 4B), indicating that *PSIG1* negatively regulates the induction of ETI-triggered cell death in an SA-independent manner.

Similarly, enhanced ROS production in response to flg22 treatment in the *psig1* mutants occurred in an SA-independent manner (Fig 2F and S5C Fig). ROS accumulation is also known to regulate the induction of cell death in both positive and negative manners [21]. Therefore, we crossed *psig1-1* with the *rbohD* mutant, which has a defect in pathogen-induced ROS production [19], and investigated the contribution of ROS accumulation to the *psig1-1* phenotypes. There was no detectable flg22-induced ROS production in the *psig1-1 rbohD* double mutant (Fig 2G and S5D Fig), and the dwarf phenotype of *psig1-1* was retained in the *rbohD* background (Fig 2D). In addition, enhanced ETI-triggered cell death phenotype of *psig1-1* was also unaffected by the *rbohD* introgression (Fig 4C and 4D and S8C Fig), indicating that *PSIG1* negatively regulates the induction of ETI-triggered cell death in a ROS-independent manner. Collectively, these results suggest that the induction of cell death by avirulent bacterial pathogen infection is limited to a certain level in WT plants, and *PSIG1* is involved in a mechanism required for the restriction of cell death in an SA- and ROS-independent manner.

We additionally investigated the contribution of SA and ROS to the *Hpa* Noco2-induced cell death. Similarly, the induction of cell death observed in the *psig1-1* mutant for cells in contact with *Hpa* Noco2 hyphae was still present in both genetic backgrounds containing *sid2-2* or *rbohD* mutations (Fig 4E). However, the induction of cell death was clearly compromised in the *sid2-2* background (Fig 4E), indicating that elevated SA levels in *psig1-1* partly contributed to the *Hpa* Noco2-induced cell death. Interestingly, we also observed a clear reduction in cell death in the *eds1-2* background (Fig 4E). This result may suggest that *Hpa* Noco2 effectors are weakly recognized by TIR-NB-LRR-type receptor proteins, and the remaining cell death is triggered by alternative NB-LRR-type receptors, or the result of reduced SA level.

### PAMP-signaling mutants display the *psig1* mutant-like cell death phenotype

**Fig 5:**
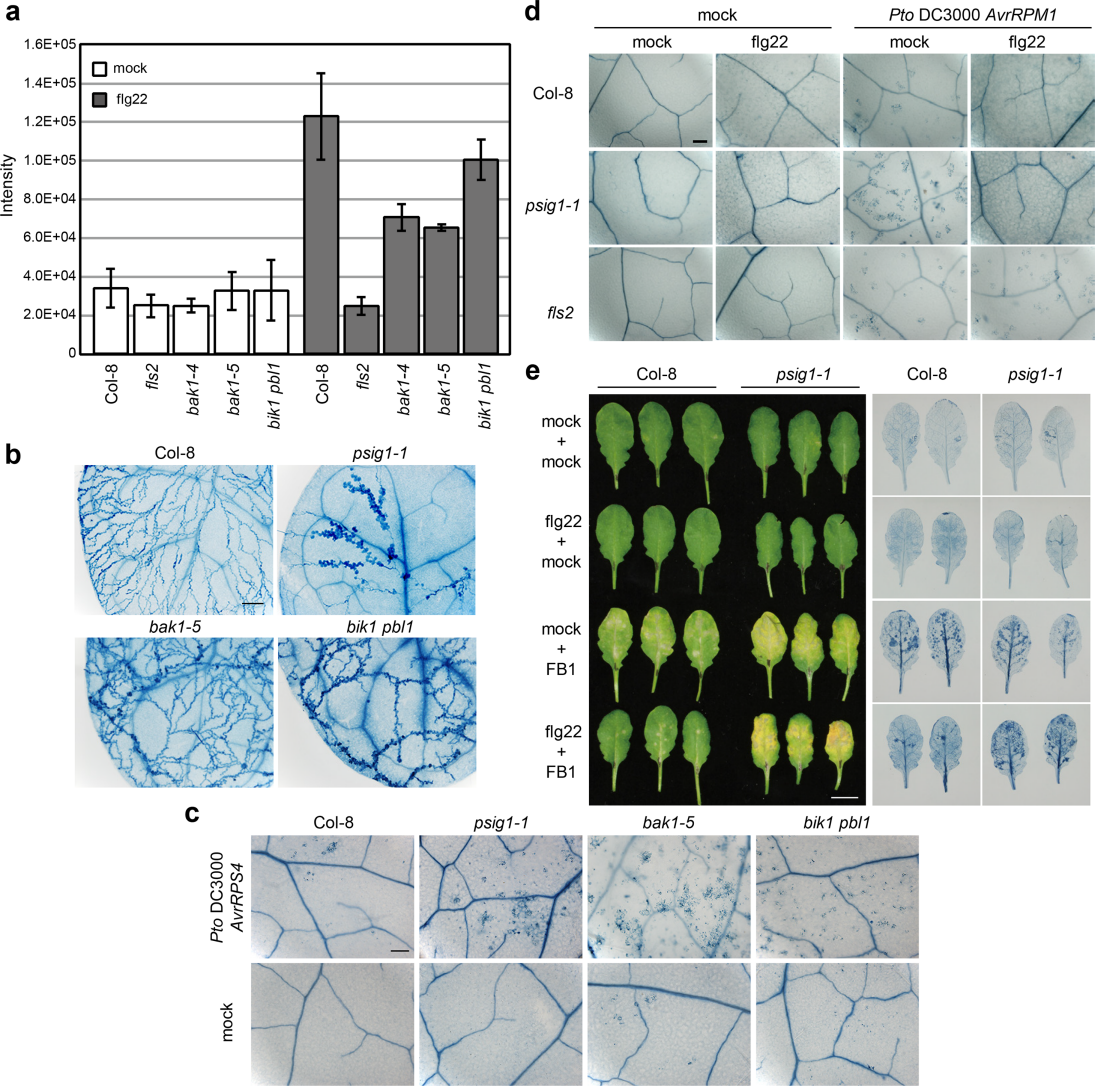
*PSIG1* is required for flg22-induced cell death suppression. **a**, Phosphoregulation of PSIG1 in the PAMP-signaling mutants. Relative abundance of the ‘DIQGSDNAIPLpSPQWLLSKPGENK’ phosphopeptide upon flg22 treatment. Arabidopsis seedlings were treated with 1 μM flg22 for 10 min or received a mock treatment (dH_2_O) prior to phosphoproteome analysis. Data are shown as the mean ± SD from three independent experiments. **b**, The *bak1-5* and *bik1 pbl1* mutants induce cell death upon *Hpa* Noco2 infection. Plants were inoculated with spores of *Hpa* Noco2, and dead cells on true leaves were visualized by trypan blue staining 5 days after inoculation. The scale bar represents 200 μm. **c**, Induction of RPS4-triggered cell death is pronounced in the *bak1-5* and *bik1 pbl1* mutants. Plants were spray inoculated with 1 x 10^8^ c.f.u. ml^-1^ of *Pto AvrRPS4*, and dead cells were visualized by trypan blue staining 2 days after inoculation. The scale bar represents 200 μm. **d**, Flg22-induced restriction of effector injection by *Pto* is intact in the *psig1-1* mutant. Leaves were infiltrated with 100 nM flg22 or received a mock treatment (dH_2_O). Twenty-four h after the pretreatments, plants were spray inoculated with 1 x 10^8^ c.f.u. ml^-1^ of *Pto AvrRPM1*, and dead cells were visualized by trypan blue staining 24 h after inoculation. The scale bar represents 200 μm. **e**, Suppression of flg22-induced FB1-triggered cell death is compromised in the *psig1-1* mutant. Leaves were infiltrated with FB1 after mock (dH_2_O) or flg22 pretreatments. Control leaves were infiltrated with dH_2_O (mock) after mock (dH_2_O) or flg22 pretreatments. Photographs were taken 4 days after FB1 infiltration. Dead cells were visualized by trypan blue staining. The scale bar represents 200μm.

As PSIG1 was originally identified as an early PAMP-responsive phosphoprotein, it is possible that PSIG1 is a PAMP-signaling component that regulates the plant immune system. If this is the case, there may be other PAMP-signaling mutants that phenocopy the *psig1* mutant cell death phenotype. BAK1 is a co-receptor of PAMP and BR receptors, and the *bak1-5* mutant is only impaired in PAMP-signaling [34]. BIK1 and PBL1 are highly homologous receptor-like cytoplasmic kinases that directly interact with PAMP receptors, and the double mutant is impaired in PAMP-induced resistance [50]. We found that flg22-induced phosphoregulation of PSIG1 was compromised in the *bak1-4*, *bak1-5*, and *bik1 pbl1* mutants (Fig 5A). In addition, the *bak1-4*, *bak1-5*, and *bik1 pbl1* mutants displayed the *psig1*-like cell death phenotype upon *Pto AvrRPS4* or *Hpa* Noco2 inoculation (Fig 5B and 5C and S10 Fig). These results suggest that activation of the PAMP-signaling pathway suppresses the induction of cell death through an elusive mechanism that is independent of the reported BAK1/SERK4-regulated cell death mechanism [30,32,33].

### PAMP-induced restriction of avirulence effector injection by bacterial pathogens is not affected in the *psig1* mutant

Activation of the PAMP-signaling pathway has been reported to inhibit HR cell death induced by avirulent bacterial pathogens by restricting the ability of type III protein secretion system to inject effectors [51]. Therefore, impaired PAMP-signaling may result in enhanced effector injection and thus excess HR cell death. To test whether *PSIG1* is involved in the PAMP-induced restriction of effector injection by *Pto*, we inoculated mutant plants with *Pto AvrRPM1*. As reported, WT plants displayed inhibition of cell death by the flg22 pretreatment, in contrast to the *fls2* mutant (Fig 5D and S11 Fig). As in WT plants, the induction of HR cell death was also inhibited in the *psig1-1* mutant by flg22 pretreatment, suggesting that the PAMP-induced restriction of effector injection by *Pto* is intact in the *psig1* mutants. These results suggest that PAMP-signaling pathways suppress effector-triggered HR cell death through at least two different mechanisms at different stages of infection.

### *PSIG1* is required for PAMP-induced suppression of necrotrophic fungal pathogen-derived mycotoxin-triggered cell death

Next, we investigated whether *PSIG1* suppresses cell death induced by different mechanisms involved in plant-pathogen interactions. Necrotrophic pathogens actively induce cell death of host cells by secreting toxins that support infections. Fumonisin B1 (FB1) is a mycotoxin produced by the necrotrophic fungal plant pathogen *Fusarium moniliforme*. FB1 induces a type of PCD with some similarities to HR cell death [47]. Of note, FB1-induced PCD can be suppressed by PAMP pretreatment in Arabidopsis [35]. FB1 treatment resulted in leaf chlorosis and cell death, which was confirmed by trypan blue staining, in both *psig1-1* and WT plants (Fig 5E). The pronounced induction of cell death in the *psig1-1* mutant is most likely the result of elevated SA levels [9]. Pretreatment with flg22 less effectively suppressed the cell death in the *psig1-1* mutant, irrespective of the enhanced FB1-triggered cell death, as compared to WT plants (Fig 5E). These results indicate that *PSIG1* participates in the PAMP signaling-dependent suppression of FB1-induced PCD.

### The GYF domain is required for the cell death regulation

**Fig 6:**
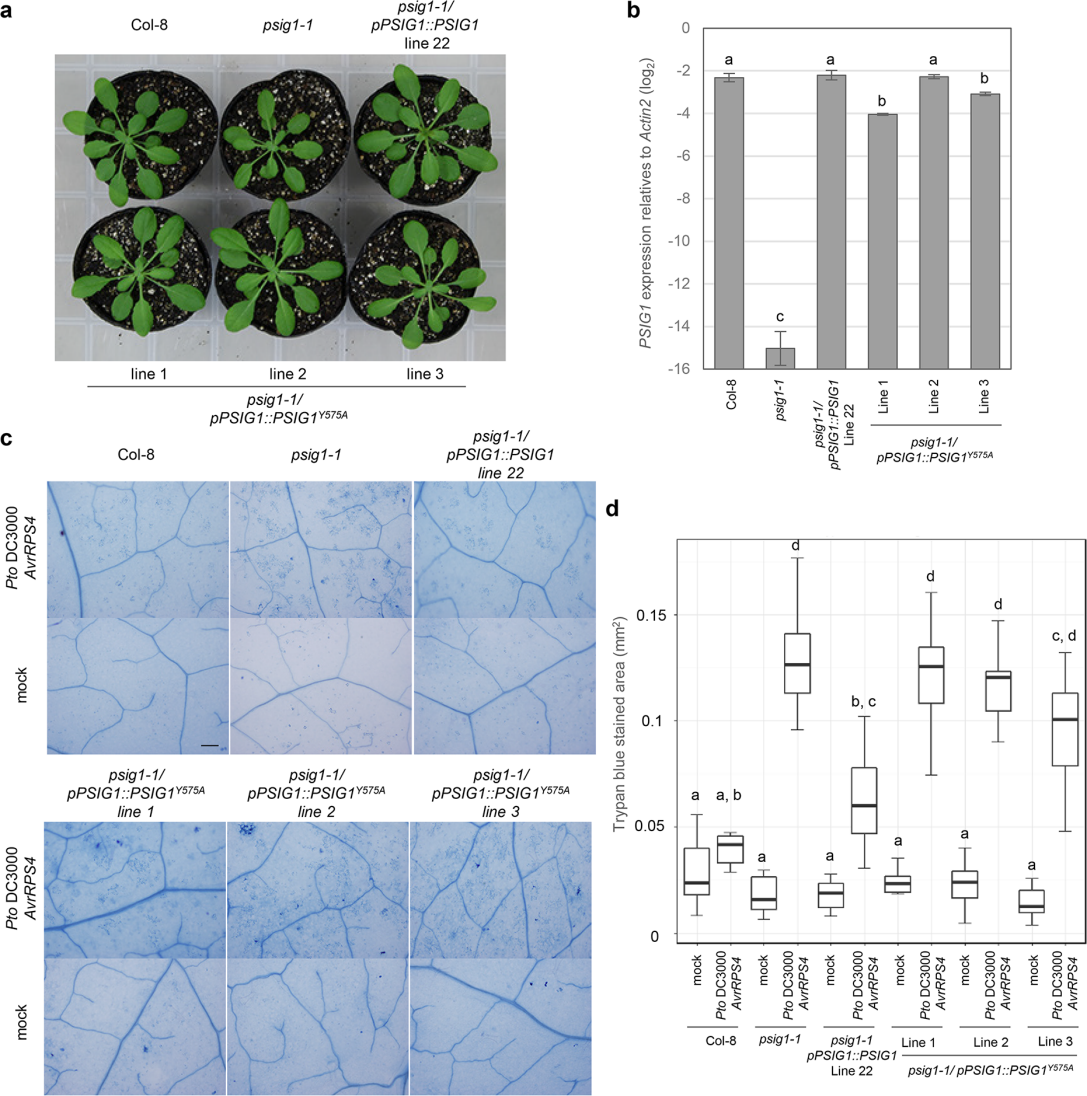
The GYF domain is required for the cell death but not growth regulation. **a**, Expression of *PSIG1*^*Y575A*^ complements the *psig1-1* growth phenotype. Photograph of 5-week-old plants grown under long day conditions (12 h light/ 12 h dark). **b**, *PSIG1* gene expression in 5-week-old plants. Data are shown as the mean ± SE. Statistical groups were determined using the Tukey HSD test. Statistically significant differences are indicated by different letters (*p* < 0.05). **c**, Plants were spray inoculated with 1 x 10^8^ c.f.u. ml^-1^ of *Pto AvrRPS4* under long day condition (12 h light/ 12 h dark), and dead cells were visualized by trypan blue staining 1 day after inoculation. The scale bar represents 200 μm. **d**, Trypan blue stained area. Plants were spray inoculated with 1 x 10^8^ c.f.u. ml^-1^ of *Pto AvrRPS4*, and dead cells were visualized by trypan blue staining 1 day after inoculation. The stained area was measured using an imaging software. Two leaves were taken from each of 4 individual plants. The box plot indicates the area of trypan blue stained cells. Boxes show upper and lower quartiles of the data, and black lines represent the medians. Statistical groups were determined using the Tukey HSD test. Statistically significant differences are indicated by different letters (*p* < 0.05).

To investigate the role of the GYF domain in the function of PSIG1, we complemented the *psig1-1* mutant with a construct driven by its native promoter and carrying Y575A mutated version of the GYF domain (Fig 6B). The Y575A mutation was shown to abrogate interaction with proline-rich sequences of interacting proteins [41,52]. Even though expression of *PSIG1*^*Y575A*^ complemented the dwarf phenotype (Fig 6A), it failed to suppress the enhanced cell death phenotype triggered by *Pto AvrRPS4* inoculation (Fig 6C and 6D). These results uncouple the GYF domain function in the regulation of cell death from other functions responsible for the dwarf phenotype. The GYF domain of PSIG1 was shown to recognize the proline-rich sequence proline-proline-glycine-phenylalanine (PPGF) [41]. Therefore, proteins that have the PPGF sequence expected to regulate the cell death along with PSIG1.

### PSIG1 localizes to P-bodies and interacts with SMG7

**Fig 7:**
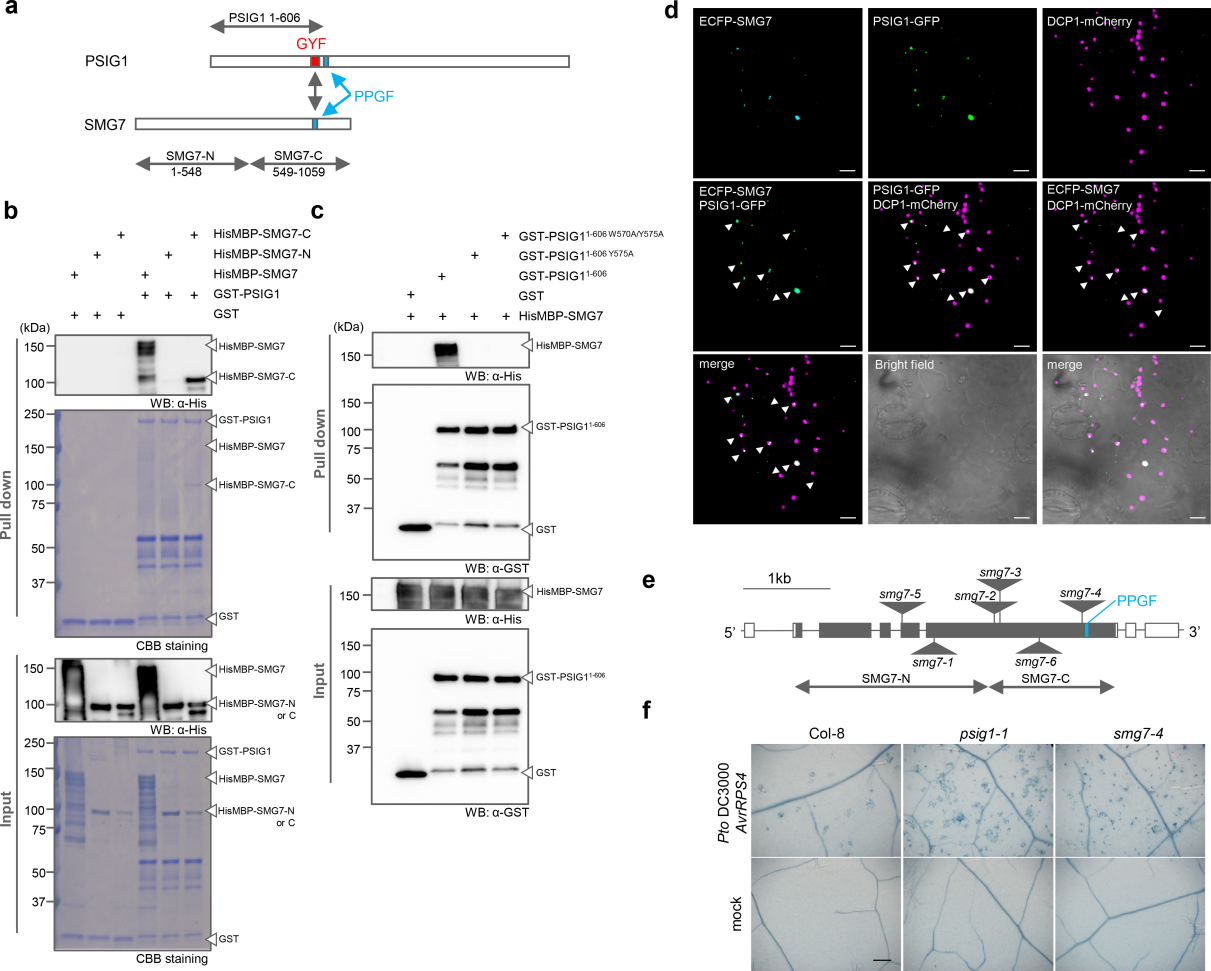
PSIG1 interacts with SMG7. **a**, Schematic structure of PSIG1 and SMG7. **b**, PSIG1 physically interacts with the C-terminus of SMG7 *in vitro*. HisMBP-SMG7, HisMBP-SMG7-N or HisMBP-SMG7-C were incubated with GST or GST-PSIG1 and the conjugates were pulled down with Glutathione-Sepharose beads. HisMBP-SMG7, HisMBP-SMG7-N, or HisMBP-SMG7-C were detected by immunoblotting using anti-His antibody. GST-PSIG1 was stained with Coomassie brilliant blue (CBB). Arrowheads indicate the position of each proteins. **c**, Tyr-575 of PSIG1 is required for interaction with SMG7 *in vitro*. HisMBP-SMG7 was incubated with GST, GST-PSIG1 ^1–606^, GST-PSIG1 ^1–606 Y575A^, or GST-PSIG1 ^1–606 W570A/Y575A^, and the conjugates were pulled down with Glutathione-Sepharose beads. HisMBP-SMG7 and GST-PSIG1 were detected by immunoblotting using anti-His antibody or anti-GST antibody. **d**, PSIG1 and SMG7 co-localize with DCP1, a P-body marker. The images show the CFP signal in cyan, the GFP signal in green, and the mCherry signal in magenta. The merged images indicate the overlay of two signals in yellow or purple and the overlay of three signals in white. White arrowheads indicate overlayed signals. Cellular localization was analyzed at 4 days after inoculation in agroinfiltrated *Nicotiana benthamiana*. The scale bars represent 10 μm. **e**, Genomic structure of the *SMG7* gene. Black boxes indicate the coding region, and white boxes indicate the non-coding region. The PPGF sequence resides downstream of the T-DNA insertion site. **f**, Induction of RPS4-triggered cell death is pronounced in the *smg7-4* mutant allele. Plants were spray inoculated with 1 x 10^8^ c.f.u. ml^-1^ of *Pto AvrRPS4*, and dead cells were visualized by trypan blue staining 2 days after inoculation. The scale bar represents 200 μm.

To explore the molecular function of PSIG1, we assessed the subcellular localization of PSIG1. The C-terminus of PSIG1 was tagged with GFP, and the labelled protein was transiently expressed in *Nicotiana benthamiana* leaves. Interestingly, PSIG1-GFP localized to cytoplasmic foci and co-localized with DCP1-mCherry, a processing body (P-body) marker (Fig 7D and S12 Fig). The GYF domain of PSIG1 was shown to recognize the proline-rich sequence PPGF [41]. Therefore, we searched for PPGF sequences in known plant P-body components and found the PPGF sequence at the C-terminal region of SMG7 (At5g19400) (Fig 7A and S13 Fig). Importantly, mutations in *SMG7* were previously shown to cause autoimmune phenotypes, including the induction of spontaneous cell death in Arabidopsis [53]. We therefore investigated whether PSIG1 interacts with SMG7. GST-tagged PSIG1 and HisMBP-tagged SMG7 recombinant proteins were prepared, and the interaction was assessed by a GST-pull down assay. We found that GST-PSIG1 interacts with HisMBP-SMG7 *in vitro* (Fig 7B). The C-terminal half of SMG7 (SMG7-C), which contains the PPGF sequence, was required for the interaction, while the N-terminal half of SMG7 (SMG7-N) was dispensable (Fig 7B). Interestingly, PSIG1 possesses the PPGF sequence (Fig 7A), opening the possibility of an self-inhibitory intramolecular interaction [41]. Although the full-length PSIG1 interacted with SMG7 *in vitro*, their interaction could be fine-tuned through the intramolecular interaction *in planta*. In spite of interacting *in vitro*, the PPGF sequence in PSIG1 could nevertheless affect the interaction with SMG7 and complicate interpretation of the results. Therefore, we decided to use the N-terminal region of PSIG1 that contains the GYF domain but not the PPGF sequence for the further analyses. To investigate the significance of the GYF domain in PSIG1 and the PPGF sequence in SMG7, we mutated residues important for the interaction [41]. Mutations in the GYF domain (Y575A and W570A/Y575A) or PPGF sequence (G933A) indeed abolished the interaction (Fig 7C and S14 Fig). Moreover, we found that PSIG1 co-localizes with SMG7 in *Nicotiana benthamiana* (Fig 7D and S12B Fig). Collectively, these results suggest that PSIG1 directly interacts with SMG7 through its GYF domain and functions within P-bodies.

### The *smg7-4* mutant allele displays a *psig1* mutant-like cell death phenotype

As in the case of the *psig1* mutants, two *smg7* mutant alleles, *smg7-4* and *smg7-6*, that have T-DNA insertions in the C-terminal region, do not show autoimmune phenotypes and are indistinguishable from WT plants (Fig 7E) [53]. The PPGF sequence that mediates interaction with the GYF domain is located downstream of the T-DNA insertion sites of *smg7-4* and *smg7-6* (Fig 7E). Therefore, if SMG7 plays a role in the regulation of PSIG1-dependent cell death, the *smg7-4* allele is expected to display the enhanced cell death phenotype as seen in the *psig1* mutants. To test this, we challenged the *smg7-4* allele with *Pto AvrRPS4* or *Pto AvrRPM1* and found that the *smg7-4* allele phenocopied the *psig1’s* enhanced cell death (Fig 7F and S15 Fig). These results suggest that PSIG1 regulates the induction of cell death by interacting via the C-terminal region of SMG7.

## Discussion

Activation of cell death during the interaction of plants with biotrophic or necrotrophic pathogens, can lead to antagonistic results [4,54]. The ability to fine-tune PCD activation during plant-pathogen interactions is critical for plants to effectively fend off pathogens with different infection strategies [3,50]. However, the biological significance of the systems regulating cell death activation remains unclear because a general framework and the molecular components of the system are poorly understood. With the initial aim of understanding PAMP-signaling pathways, we identified PSIG1 as an early PAMP-responsive protein by differential phosphoproteome analysis (S4 Table). Through our approach, we were also able to detected phospho-regulation of MAPK cascade components, SERK2, CDPKs, MPK1, RbohD, and WRKY1 (S4 Table). By isolating T-DNA insertion mutants of *PSIG1*, we found that *PSIG1* was required for resistance against the hemi-biotrophic bacterial pathogen *Pto* (Fig 3B). However, detailed analyses with well-described PAMP responses in the *psig1* mutants, did not reveal major differences to WT plants. Instead, we discovered that the induction of cell death was accelerated in the *psig1* mutants upon inoculation with avirulent bacterial pathogens (Fig 4 and S8 Fig). This observation led us to hypothesize that PSIG1 has a role in adjusting the induction of ETI-triggered cell death by potentially monitoring the active state of PTI.

The significance of SA and ROS pathways in the regulation of cell death has been well documented. However, our genetic analyses with SA biosynthesis- and ROS production-deficient mutants indicated that *PSIG1* regulates the induction of cell death in an SA- and ROS-independent manner (Fig 4A–4D). In this respect, the *psig1* mutants differ strikingly from the *lsd1* and autophagy mutants that require SA accumulation for their uncontrolled PCD phenotypes [11,26,55], suggesting that *PSIG1* controls cell death through an alternative pathway.

Moreover, we addressed whether *PSIG1* regulates the induction of cell death in a PAMP signal-dependent manner. Up to now, there has been no experimental system available to evaluate effect of PTI on the induction of HR cell death [35]. Therefore, we focused on the inhibitory effect of PTI on PCD induction by the fungal toxin FB1 [35], and reveal that the PTI-induced inhibition was compromised in the *psig1* mutant (Fig 5E). Collectively, these results suggest that plants possess a pathway to restrict cell death induction during PTI. A potential reason may be to override cell death activation during an effective PTI response to avoid unnecessary tissue damage and infection by necrotrophic pathogens. It will be important to address how *PSIG1* suppresses the cell death triggered by ETI activation and the fungal toxin FB1. To that purpose, however, molecular mechanisms how ETI and FB1 lead to the cell death must be unraveled.

With regards to the enhanced cell death phenotype of the *psig1* mutants, they expectedly displayed enhanced resistance against the biotrophic pathogen *Hpa* Noco2, and enhanced susceptibility to the necrotrophic pathogen *Alternaria brassiciola* O-264 (Fig 3G). Necrotrophic pathogens could kill host cells in a variety of ways, some of which may not utilize PCD machinery of host cells. Therefore, the *psig1* mutants may not necessarily display susceptible phenotype against all necrotrophic pathogens. Again, an intriguing observation was that the *psig1* mutants were more susceptible to the hemi-biotrophic pathogen *Pto* compared to WT plants. These results suggest that the PTI-dependent PCD regulation system may be crucial for coping with hemi-biotrophic pathogens that can switch their infection strategies during colonization. In addition, we found that resistance against the avirulent bacteria strains was not enhanced in the *psig1* mutants (Fig 3D and S17 Fig), supporting the idea that the host plants do not benefit much by maximizing the induction of HR cell death. Taken together, these results have allowed us to identify PSIG1 as a unique component in plant immunity that connects PTI and PCD, highlighting the biological significance of the PTI-dependent PCD regulation. Our findings may also offer an explanation why toxin-producing necrotrophic pathogens potentially suppress PTI for their successful infection [56].

The *psig1* mutants, especially the *psig1-1* and *psig1-2* alleles, seem to be sensitive to environmental conditions. Recently, *exa1-1/muse11-1/psig1-1* was reported to display auto-immune phenotype, which was characterized by elevated *PR1* gene expression and reduced plant size [43]. However, these phenotypes were not observed in another study [36]. Under our experimental conditions, the *psig1-1* allele displays intermediate phenotype based on the *PR1* gene expression and plant size. Importantly, the *psig1-3* allele displays the cell death phenotype but is less sensitive to growth conditions compared to the *psig1-1* and *psig1-2* alleles. The difference between these alleles may result from the expression of a C-terminally truncated PSIG1 in *psig1-3* allele (S2C Fig). The *psig1-3* allele is a useful tool to assess significance of the cell death regulation in disease resistance. Indeed, the *psig1-3* allele clearly displayed susceptible phenotype against *Pto* also when it was inoculated by syringe infiltration, while the *psig1-1* and *psig1-2* alleles gave inconsistent results (S16 Fig). The *psig1-1* and *psig1-2* alleles are more likely to be affected by stress responses caused by the syringe infiltration. Interestingly, the *psig1-3* allele became susceptible 3 days post inoculation (dpi) but displayed no difference 2 dpi compared to WT plants, which is different from the *eds1-2* mutant that displayed susceptible phenotype 2 dpi (S16B Fig). A similar trend was observed by spray inoculation (S5B Fig). In contrast, growth of the avirulent strain *Pto AvrRPS4* was unaffected in the *psig1* mutants (S17 Fig). These observations, especially the *Pto* growth kinetics in the *psig1-3* allele, support our hypothesis that the cell death regulation becomes crucial when hemi-biotrophic pathogens switch their infection strategies.

The GYF domains found in plant proteins are all classified into the SMY2-type GYF domain family (S1 Fig). Although the structure and recognition properties of the GYF domains in plant proteins are highly conserved, the overall structure of GYF domain-containing proteins in plants is rather plant specific (Fig 1D, S1B Fig and S1 and S2 Tables) [41]. *PSIG1* gene family is well conserved in land plants but not in other organisms. No additional domains with known function are predicted on PSIG1, which would help elucidate further molecular function(s). In many cases, the GYF domain proteins modulate signaling pathways through their binding to key regulatory components. Therefore, identification of PSIG1 interactors are expected to facilitate an understanding of the molecular mechanisms by which PSIG1 inhibits PCD. A previous study identified the PPGF sequence containing fragments of RNA binding proteins in a Y2H screen using the GYF domain of PSIG1 as a bait [41]. Interestingly, PSIG1 itself has the PPGF sequence (Fig 7A), and the intramolecular interaction could happen and affect interaction with other PPGF sequence containing proteins [41]. In another study, PSIG1 was identified in 5′-cap complexes [57]. These findings suggest that PSIG1 might be involved in regulating RNA metabolism and/or translation.

Indeed, we found that PSIG1 localizes to P-bodies that are involved in post-transcriptional regulation [58] (Fig 7D). Moreover, we found that PSIG1 interacts with the NMD factor SMG7 through the GYF domain (Fig 7C). In general, SMG7 is functionally conserved among different organisms [53,59,60]. However, the PPGF sequence recognized by the GYF domain was only found in the less conserved C-terminal region of plant SMG7 homologs (S13A Fig). Additionally, the GYF domain of yeast Smy2 was shown to interact with P-body components other than the SMG7 homolog [61]. These results suggest that the GYF domain proteins function within P-bodies in general but cooperate with different components in different organisms.

Disruption of the NMD-related genes often results in autoimmune phenotypes [62]. In addition, PAMP treatment was shown to modulate NMD efficiency [63]. Moreover, the C-terminal region of SMG7 was suggested to play a role in the target degradation step of plant NMD [64]. The observations that PSIG1 interacts with the C-terminal region of SMG7 (Fig 7B) and that the C-terminal truncated *smg7-4* allele phenocopies the *psig1* cell death phenotype (Fig 7F) suggest that PSIG1 regulates the induction of cell death by modulating an SMG7-dependent NMD. It will be important to identify PSIG1- and SMG7-dependent NMD targets to understand how NMD contributes to the induction of cell death.

## Materials and methods

### Plant materials and growth conditions

Arabidopsis plants were grown in soil at 22°C in 8 h light /16 h dark or 12 h light /12 h photoperiods. Mutant seeds for *psig1-1* (SALK_005994), *psig1-2* (SALK_135013), *psig1-3* (SAIL_1282_B9), *smg7-4* (SAIL_63F08) were obtained from the Nottingham Arabidopsis Stock Center (NASC). Homozygous insertion mutants were identified by PCR. The *fls2* (SALK_141277), *rbohD* [19] and *sid2-2* [45] seeds were kindly provided by Kohki Yoshimoto (Meiji University, Japan). The seeds for the null *eds1-2* mutation in the Col-0 background (referred to as Col *eds1-2*) [65] were kindly provided by Shigeyuki Betsuyaku (University of Tokyo, Japan) and Jane Parker (MPI for Plant Breeding Research, Germany). The *bik1 pbl1* [44], *bak1-4* (SALK_116202) and *bak1-5* [34] seeds were kindly provided by Cyril Zipfel (The Sainsbury Laboratory, UK) and Jian-Min Zhou (National Institute of Biological Sciences, Beijing, China). Double mutants were generated by crossing individual mutants and were identified by PCR. Primer sets used in this study are listed in S3 Table.

### Phylogenetic analysis

Phylogenetic analysis was performed using MEGA6.06 software (www.megasoftware.net). Full-length amino acid sequences were used to generate the phylogenetic tree. The bootstrap value was set at 10,000 replicate samples.

### Phosphoproteome analysis

Arabidopsis seedlings were grown in liquid MGRL medium with 0.1% (w/v) sucrose [66] at 22°C under continuous light for 10 days. Phosphoproteome analysis was performed as described previously with minor modifications [67]. MS peaks were detected using an in-house 2DICAL software package [68] that identified all MS/MS spectra using Mascot software, adjusted the retention times of each LC-MS data point utilizing the similarity index of the mass spectrum pattern, and grouped peaks from different samples that were derived from same peptides in the direction of acquiring time. The peak intensities of MS chromatograms were used for quantitative values. Three biological replicates were analyzed for each condition, and the significance of differences was tested. Statistical relevance was determined using a two-tailed Student’s *t*-test (*p*-value < 0.05).

### RNA isolation and qRT-PCR analysis

Total RNA was isolated using the RNeasy Plant Mini Kit (Qiagen, Netherlands), and cDNA was prepared using the ReverTra Ace Reverse Transcription Kit (Toyobo, Japan). Quantitative reverse transcription PCR (qRT-PCR) was performed using the Mx3000P QPCR system (Agilent Technologies, USA) with the Thunderbird SYBR qPCR Mix (Toyobo, Japan). Data were analyzed using an in-house script written in the R language (S1 Information) as described previously [69].

### ROS assay

Arabidopsis seedlings were grown in liquid MGRL medium with 0.1% (w/v) sucrose [66] at 22°C under continuous light for 10 days. Ten 10-day-old Arabidopsis seedlings were incubated in liquid MGRL medium supplemented with 0.1% (w/v) sucrose containing 100 μM 8-amino-5-chloro-7-phenylpyrido [3,4-d] pyridazine-1,4-(2H,3H) (L-012) (Wako, Japan) for 2 h at 22°C under darkness, followed by transfer to liquid MGRL medium containing 100 nM flg22, a peptide that perceives bacterial flagellin. ROS production was determined by counting photons derived from L-012–mediated chemiluminescence using NightSHADE LB985 (Berthold Technologies, Germany).

### MAPK assay

Arabidopsis seedlings were grown in liquid MGRL medium with 0.1% (w/v) sucrose [66] at 22°C under continuous light for 10 days. Proteins were extracted from 100 nM flg22-treated or mock-treated seedlings in extraction buffer (50 mM Tris-HCl (pH 7.5), 10 mM MgCl_2_, 15 mM EGTA, 100 mM NaCl, 2 mM DTT, 1 mM sodium fluoride, 0.5 mM Na_3_VO_4_, 30 mM β-glycerophosphate, 0.1% (v/v) NP-40 and one Complete tablet, EDTA-free per 50 ml (Roche, Germany)). Phosphorylated MAPK proteins were detected by immunoblot analysis with anti-phospho-p44/42 MAPK (Erk1/2) (Thr202/Tyr204) (D13.14.4E) rabbit mAb (Cell Signaling Technology, USA) [61]. The blotted membrane was stained with Coomassie Brilliant blue (CBB) to verify equal loading.

### Callose deposition assay

The callose deposition assay was performed as described previously with minor modifications [70]. Leaves from 6-week-old plants were syringe infiltrated with 100 nM flg22 or 10 mM MgCl_2_. Leaf discs were removed 20 h post-infiltration using an 8-mm-diameter cork borer and fixed in an acetic acid:ethanol (1:3) solution for several h. Leaves were rehydrated in 70% (v/v) ethanol for 2 h, 50% (v/v) ethanol for 2 h, and overnight in water. Leaves were stained for 1 h in the dark in 150 mM sodium phosphate buffer (pH 9.0) containing 0.05% (w/v) aniline blue. Leaves were mounted in 10% glycerol (v/v) and the localization of fluorescently stained callose was determined by fluorescence microscopy. Callose deposition was quantified by Image-J software (http://imagej.nih.gov/ij/). At least three leaves from three independent plants were used as biological replicates for the analysis.

### Pathogen infection assays

*Pseudomonas syringae* pv. *tomato* DC3000 (*Pto*), *Pto AvrRPM1* or *Pto AvrRPS4* were grown on King’s B medium at 28°C for 2 to 3 days. Six to 7-week-old plants grown in the 8 h light /16 h photoperiod were spray inoculated with bacterial suspensions of 5 x 10^7^ to 5 x 10^8^ c.f.u. (colony forming unit) ml^-1^ containing 0.04% silwet-L77 (Bio medical science, Japan). For the infiltration assay, 5 to 6-weeks-old plants grown in the 12 h light /12 h photoperiod were syringe infiltrated with bacterial suspension of 5 x 10^4^ c.f.u. ml^-1^. For bacterial growth experiments, inoculated plants were maintained at high humidity at 22°C in the 8 h light /16 h dark or 12 h light /12 h photoperiods. *Hyaloperonospora arabidopsidis* isolate Noco2 was maintained weekly by transferring spores onto WT Col-8 plants growing at 16°C in an 8 h light /16 h dark photoperiod. Fourteen-day-old plants were spray inoculated with 5 x 10^4^ spores ml^-1^ of *Hpa* Noco2. Infected plants were maintained at high humidity at 16°C in an 8 h light /16 h dark photoperiod. Infection development was scored 6 days after infection by counting sporangiophores on true leaves. *Alternaria brassicicola* isolate O-264 was maintained on potato dextrose agar medium. The third to fourth leaves of 27-day-old plants were inoculated with 5 μl drops of a spore suspension (5 x 10^5^ spores ml^-1^ in distilled water). Infected plants were maintained at high humidity at 16°C in an 8 h light /16 h dark photoperiod. Lesion sizes were measured at 6 days after inoculation using Image-J software (http://imagej.nih.gov/ij/).

### Trypan blue staining

Trypan blue staining was performed as described previously with minor modifications [71]. Leaves were stained in lactophenol-trypan blue solution (10 mg trypan blue dissolved in 80 ml of lactic acid:glycerol:phenol:distilled water:ethanol 1:1:1:1:4, v/v) and cleared in chloral hydrate solution (2.5 g chloral hydrate in 1 ml distilled water). Leaves were mounted in chloral hydrate solution and dead cells were observed using a BX51 microscope (Olympus, Japan). Trypan blue staining area was measured using Photoshop CC software (Adobe, USA). Statistical analysis was performed using the Tukey HSD test (S2 Information) with R software version 3. 2. 3 (https://www.r-project.org/).

### Fumonisin B1 assay

The fumonisin B1 assay was performed as described previously with minor modifications [35]. Leaves from 6 to 7-week-old plants were syringe infiltrated with 100 nM flg22 or distilled water (mock) 24 h prior to FB1 treatment. Pretreated leaves were then syringe infiltrated with 50 μM FB1 (Sigma-Aldrich, USA) or a mock solution (distilled water containing 1% (v/v) methanol) and plants were kept at 22°C in a 16 h light /8 h dark photoperiod. Photographs were taken 4 days after FB1 treatment, and leaves were stained with trypan blue to visualize dead cells.

### Constructs and transformation

The open reading frame (ORF) of *PSIG1* was cloned into pENTR4 dual-selection vector (Thermo Fisher SCIENTIFIC, USA) between *Sal*I and *Not*I sites using an IN-FUSION HD Cloning Kit (Clontech Laboratories, USA). The 1.5 kb promoter fragment of *PSIG1* was cloned into the *Sal*I site of the pENTR4-PSIG1 construct using IN-FUSION HD Cloning Kit. *PSIG1pro*::*PSIG1* stop codon deletion and *PSIG1pro*::*PSIG1*^*Y570A*^ were generated by PCR-based mutagenesis using the *PSIG1pro*::*PSIG1* construct as a template. *PSIG1pro*::*PSIG1* and *PSIG1pro*::*PSIG1*^*Y570A*^ were subcloned into binary vector pGWB1[72], and the stop codon-mutated *PSIG1pro*::*PSIG1* was subcloned into pGWB4 using LR clonase II enzyme mix (Thermo Fisher SCIENTIFIC, USA) [64]. The binary vectors were introduced into *Agrobacterium tumefaciens* C58C1. The *psig1-1* mutant was transformed using a floral dip method [73]. T3 or T4 homozygous transformants were used in this study.

### Recombinant protein purification

The *PSIG1* ORF was amplified by PCR and cloned into pGEX4T-3 vector (GE Healthcare) between *Sal*I and *Not*I sites. PSIG1^1-606^, not including its own PPGF motif, was cloned into the pGEX4T-3 vector between *Sal*I and *Not*I sites. GST-PSIG1^1-606 Y575A^ and GST-PSIG1^1-606 W570A/Y575^ were produced by PCR-based mutagenesis using PrimeSTAR Mutagenesis Basal Kit (TaKaRa, Japan). The *Nco*I site of the pENTR4 dual selection vector was mutagenized with the PrimeSTAR Mutagenesis Basal Kit to avoid the translation of additional amino acids from the *Nco*I site and was named pENTR4m. The open reading frame of AtSMG7, AtSMG7-N (1–548) and AtSMG7-C (549–1059) were amplified by PCR from Arabidopsis seedling (Col-8) cDNA and cloned into the pENTR4m vector between *Sal*I and *Not*I sites using the IN-FUSION HD Cloning Kit. AtSMG7^G933A^ were produced by PCR-based mutagenesis using PrimeSTAR Mutagenesis Basal Kit. AtSMG7, AtSMG7-N, AtSMG7-C and AtSMG7^G933A^ were subcloned into pDEST-HisMBP vector [74] using the LR clonase II enzyme mix. GST-PSIG1, GST-PSIG1^1-606^, GST-PSIG1^1-606 Y575A^, GST-PSIG1^1-606 W570A/Y575^, HisMBP-SMG7, HisMBP-SMG7-N, HisMBP-SMG7-C and HisMBP-SMG7^G933A^ were expressed in *Escherichia coli* Rosetta-gami^TM^ 2 (DE3) (Merck Millipore, Germany) and were purified with Glutathione-Sepharose B columns (GE Healthcare) for GST-tagged proteins or Dextrin-Sepharose High Performance columns (GE Healthcare) for MBP-tagged proteins.

### GST-pull down assay

The purified recombinant PSIG1 and SMG7 proteins were mixed in a binding buffer [50mM Tris-HCl pH7.5, 150 mM NaCl, 1 mM dithiothreitol (DTT), 0.1% (v/v) Triton X-100 and one Complete tablet, EDTA-free per 50 mL (Roche)]. The Glutathione-Sepharose B beads were mixed with the protein mixtures and incubated for 2 h at 4°C. The beads were washed three times with a washing buffer [50mM Tris-HCl pH7.5, 300 mM NaCl, 1 mM dithiothreitol (DTT), 0.1% (v/v) Triton X-100 and one Complete tablet, EDTA-free per 50 mL (Roche)]. Proteins were eluted from the beads with 2 x SDS buffer, and were detected by SDS-PAGE or immunoblotting.

### Localization analysis

A *Nicotiana benthamiana* transient expression system was used for subcellular localization analysis. The stop codon-mutated *PSIG1* ORF (*PSIG1sm*) was amplified by PCR and cloned into the pENTR4m between *Sal*I and *Not*I sites using the IN-FUSION HD Cloning Kit. The *PSIG1* and *PSIG1sm* fragments were subcloned into pGWB6 and pGWB5 binary vectors, respectively, using the LR clonase II enzyme mix. The *SMG7* fragment was subcloned into pGWB45 or pGWB661 binary vectors [75]. The P-body marker construct pTA7002::DCP1-mCherry was described previously [76]. The binary vectors were introduced into *A*. *tumefaciens* C58C1 strain carrying pCH32 plasmid. Agrobacterium cultures harboring each construct were incubated overnight at 28°C and subsequently resuspended in 10 mM MES pH 5.6, 10 mM MgCl_2_ buffer with 100 μM acetosyringone at an optical density of 0.3 at 600 nm. Agrobacterium cultures were mixed and syringe infiltrated into *N*. *benthamiana* leaves. Agrobacterium carrying pJL3-p19 was co-infiltrated [69]. Two days after inoculation, agroinfiltrated leaves were sprayed with 30 μM dexamethasone (Dex). One day after Dex treatment, localization was observed using Leica TCS SP5II (Leica Microsystems, Germany).

### Database search

Yeast and human SMG7 homologs were described by Reichenbach, P. *et al*. 2003 [77]. SMG7 homologs in plants were identified using the Phytozome database (https://phytozome.jgi.doe.gov/pz/portal.html): *Arabidopsis thaliana* TAIR10, *Chlamydomonas reinhardtii* v5.5, *Oryza sativa* v7_JGI, *Physcomitrella patens* v1.6 and *Selaginella moellendorffii* v1.0.

## Supporting information

S1 Fig The GYF domain proteins. **a**, Aligned amino acid sequences of GYF domains from diverse eukaryotic organisms. Key residues for GYF domains are delineated as white text on a black or gray background. The conserved Ile residue of Ile-type GYF domains are indicated in bold text. At, Os, Smo, Phpat, Cre, Kfl, Hs, and Sc stand for following species: *Arabidopsis thaliana*, *Oryza sativa*, *Selaginella moellendorffii*, *Physcomitrella patens*, *Chlamydomonas reinhardtii*, *Klebsormidium flaccidum*, *Homo sapiens*, and *Saccharomyces cerevisiae*, respectively. **b**, Phylogenetic tree and schematic structure of GYF-domain proteins from diverse eukaryotic species. Species abbreviations are defined in S1A Fig. Numbers on the phylogenetic tree indicate the bootstrap values. Red boxes indicate the GYF domain. Green, orange and blue boxes indicate the SWIB/MDM2 domain, the Plus-3 domain and a zinc-finger domain, respectively.
(TIF)

S2 Fig The *psig1* T-DNA insertion mutants. **a**, The *psig1* mutant alleles display a slight dwarf phenotype. Photograph of 6-week-old plants grown under short day conditions. **b**, Genomic structure of the *PSIG1* gene and the position of the T-DNA insert. Exons are indicated as black boxes. The T-DNA insertion sites are indicated by grey triangles. Black arrowheads indicate the gene-specific primer sets used for *PSIG1* gene expression analysis. **c**, *PISG1* gene expression in the *psig1* mutants. Semi-quantitative RT-PCR was performed using specific primer sets as indicated in b. The *Actin1* gene was used as an internal control.
(TIF)

S3 Fig Flg22 responses in the *psig1* mutants. **a**, Flg22-induced ROS production in the *psig1* mutants. Data are shown as the mean ± SE. **b**, Flg22-induced MAPK activation in the *psig1* mutants. **c**, Flg22-induced callose deposition in the *psig1* mutants. The scale bar represents 200 μm. **d**, Callose deposition was quantified with Image J software. Data are shown as the mean ± SE. Statistical groups were determined using the Tukey HSD test. Statistically significant differences are indicated by different letters (*p* < 0.05).
(TIF)

S4 Fig *PR1* gene expression in the *psig1* mutants. **a**, *PR1* gene expression in 10-day-old liquid culture grown seedlings. Expression data for *PR1* are shown as the mean ± SE. Statistical groups were determined using the Tukey HSD test. Statistically significant differences are indicated by different letters (*p* < 0.05). **b**, *PR1* gene expression in leaves of soil grown plants. Expression data for *PR1* gene are shown as the mean ± SE. Six-week-old plants were syringe infiltrated with 1 x 10^6^ c.f.u. of *Pto*. Statistical groups were determined using the Tukey HSD test. Statistically significant differences are indicated by different letters (*p* < 0.05).
(TIF)

S5 Fig The *psig1* mutant phenotypes. **a** and **b**, The *psig1* mutants were more susceptible to *Pto*. Plants were spray inoculated with 1 x 10^8^ c.f.u. ml^-1^ of *Pto*, and bacterial growth was determined at 0 and 2 or 3 dpi. Data are shown as the mean ± SE. Statistical groups were determined using the Tukey HSD test. Statistically significant differences are indicated by different letters (*p* < 0.05). **c** and **d**, Flg22-induced ROS production in the *psig1-1 sid2-2* and *psig1-1 rbohD* mutants. These graphs show the result of ROS production by 100 nM flg22 treatment in Arabidopsis seedlings in these genetic backgrounds.
(TIF)

S6 Fig Phenotypes of complemented lines. **a**, Photograph of 7 week-old plants grown under short day conditions. **b**, *PSIG1* gene expression in 6-week-old plants. Data are shown as the mean ± SE. Statistical groups were determined using the Tukey HSD test. Statistically significant differences are indicated by different letters (*p* < 0.05). **c**, Flg22-induced ROS production. Data are shown as the mean ± SE. **d**, *Pto* infection. Plants were spray inoculated with 1 x 10^8^ c.f.u. ml^-1^ of *Pto*, and bacterial growth was determined at 0 and 3 dpi. Data are shown as the mean ± SE. Statistical groups were determined using the Tukey HSD test. Statistically significant differences are indicated by different letters (*p* < 0.05). **e**, Photograph of *Hpa* Noco2-infected leaves. Plants were inoculated with *Hpa* Noco2, and the true leaves were stained with trypan blue 6 days after inoculation. The scale bar represents 200 μm. **f**, *Hpa* Noco2 infection. Fourteen-day-old seedlings were inoculated with spores of *Hpa* Noco2, and the number of sporangiophores was scored (0 = 1, 1–10 = 2, 11–20 = 3, >20 = 4) on true leaves 6 days after inoculation. Bars show the percentage of leaves for each score (n>25).
(TIF)

S7 Fig Stomatal density and stomatal closure response. **a**, Flg22-induced stomatal closure in the *psig1*-*1* mutant. Stomatal apertures were measured following treatment with 5 μM flg22 or mock (dH_2_O) for 60 min. Data are shown as the mean ± SE (n = 24). Statistical groups were determined using the Tukey HSD test. Statistically significant differences are indicated by different letters (*p* < 0.05). **b**, Stomatal density. Boxplots represent stomatal density (n = 36). Boxes show upper and lower quartiles of the data, and black lines represent the medians. No significant difference was observed between *psig1-1* and Col-8 according to the Student t-test (*p* < 0.05).
(TIF)

S8 Fig The *psig1* mutants displayed enhanced cell death upon infection with *Pto AvrRPM1* or *Pto AvrRPS4*. **a, c, d** and **f**, Plants were spray inoculated with 1 x 10^8^ c.f.u. ml^-1^ of *Pto AvrRPM1* or *Pto AvrRPS4*, and dead cells were visualized by trypan blue staining 1 or 2 day after inoculation. The scale bar represents 200 μm. **b, e** and **g**, Trypan blue stained area. Plants were spray inoculated with 1 x 10^8^ c.f.u. ml^-1^ of *Pto AvrRPM1* or *Pto AvrRPS4*, and dead cells were visualized by trypan blue staining 1 or 2 day after inoculation. The stained area was measured using an imaging software. Two leaves were taken from each of 2 individual plants for mock treatment. Three leaves were taken from each of 3 individual plants for pathogen treatment. The box plot indicates the area of trypan blue stained cells. Boxes show upper and lower quartiles of the data, and black lines represent the medians. Statistical groups were determined using the Tukey HSD test. Statistically significant differences are indicated by different letters (*p* < 0.05).
(TIF)

S9 Fig The *psig1 eds1-2* (Col) mutant displayed enhanced cell death upon infection with *Pto AvrRPM1*. **a**, Plants were dip inoculated with 1 x 10^8^ c.f.u. ml^-1^ of *Pto AvrRPM1*, and dead cells were visualized by trypan blue staining 1 day after inoculation. The scale bar represents 200 μm. **b**, Trypan blue stained area. Plants were spray inoculated with 1 x 10^8^ c.f.u. ml^-1^ of *Pto AvrRPM1*, and dead cells were visualized by trypan blue staining 1 day after inoculation. The stained area was measured using an imaging software. Two leaves were taken from each of 2 individual plants. The box plot indicates the area of trypan blue stained cells. Boxes show upper and lower quartiles of the data, and black lines represent the medians. Statistical groups were determined using the Tukey HSD test. Statistically significant differences are indicated by different letters (*p* < 0.05).
(TIF)

S10 Fig Cell death induction in the *bak1-4* mutant. **a**, Plants were inoculated with *Hpa* Noco2 spores, and dead cells on the true leaves were visualized by trypan blue staining 6 days after inoculation. The scale bar represents 200 μm. **b**, Plants were spray inoculated with 1 x 10^8^ c.f.u. ml^-1^ of *Pto AvrRPS4*, and dead cells were visualized by trypan blue staining 2 days after inoculation. The scale bar represents 200 μm. **c**, Trypan blue stained area. Plants were spray inoculated with 1 x 10^8^ c.f.u. ml^-1^ of *Pto AvrRPS4*, and dead cells were visualized by trypan blue staining 2 days after inoculation. The stained area was measured using an imaging software. Two to 3 leaves were taken from each of 3 individual plants. The box plot indicates the area of trypan blue stained cells. Boxes show upper and lower quartiles of the data, and black lines represent the medians. Statistical groups were determined using the Tukey HSD test. Statistically significant differences are indicated by different letters (*p* < 0.05).
(TIF)

S11 Fig *Pto AvrRPM1* induced cell death was suppressed by pre-treatment of flg22 in *psig1-1*. Trypan blue stained area. Pretreated plants either with 1 μM flg22 or dH_2_O (mock) were spray inoculated with 1 x 10^8^ c.f.u. ml^-1^ of *Pto AvrRPM1*, and dead cells were visualized by trypan blue staining 1 day after inoculation. The stained area was measured using an imaging software. Three leaves were taken from each of 3 individual plants. The box plot indicates the area of trypan blue stained cells. Boxes show upper and lower quartiles of the data, and black lines represent the medians. Statistical groups were determined using the Tukey HSD test. Statistically significant differences are indicated by different letters (*p* < 0.05).
(TIF)

S12 Fig PSIG1 is associated with DCP1 and SMG7. The images show the GFP signal in green and the mCherry or TagRFP signal in red. The merged images indicate the overlay of two signals in yellow. **a**, Subcellular localization was analyzed at 4 days after inoculation in agroinfiltrated *Nicotiana benthamiana*. **b**, Subcellular localization was analyzed at 3 days after inoculation in agroinfiltrated *N*. *benthamiana*. Before analysis, *N*. *benthamiana* was incubated at 37°C for 30 min. The scale bar represents 10 μm.
(TIF)

S13 Fig Plant SMG7 homologs have the PPGF sequence in the non-conserved C-terminal region. **a**, Phylogenetic tree and schematic structures of SMG7 from diverse eukaryotic organisms. Numbers on the phylogenetic tree indicate the bootstrap values. Dark blue boxes indicate the EST1 domain. Yellow, green and blue boxes indicate the Est1 DNA/RNA binding domain, the TPR domain and the PPGF motif, respectively. At, Os, Smo, Pp, Cre, Ce, Hs and Sc stand for following species: *Arabidopsis thaliana*, *Oryza sativa*, *Selaginella moellendorffii*, *Physcomitrella patens*, *Chlamydomonas reinhardtii*, *Caenorhabditis elegans*, *Homo sapiens* and *Saccharomyces cerevisie*, respectively. **b**, Aligned amino acid sequences around the PPGF sequence. The PPGF sequence is delineated as white text on a black background.
(TIF)

S14 Fig The PPGF sequence of SMG7 is required for interaction with PSIG1 *in vitro*. HisMBP-SMG7 or HisMBP-SMG7^G933A^ was incubated with GST, GST-PSIG1^1-606^, or GST-PSIG1^1-606 Y575A^, and the conjugates were pulled down with Glutathione-Sepharose beads. HisMBP-SMG7 and GST-PSIG1 were detected by immunoblotting using anti-His antibody or anti-GST antibody.
(TIF)

S15 Fig The *smg7-4* mutant allele displayed enhanced cell death upon infection with *Pto AvrRPM1*. **a**, Plants were spray inoculated with 1 x 10^8^ c.f.u. ml^-1^ of *Pto AvrRPM1*, and dead cells were visualized by trypan blue staining 1 day after inoculation. The scale bar represents 200 μm. **b** and **c**, Trypan blue stained area. Plants were spray inoculated with 1 x 10^8^ c.f.u. ml^-1^ of *Pto AvrRPM1* or *Pto AvrRPS4*, and dead cells were visualized by trypan blue staining 1 or 2 day after inoculation. The stained area was measured using an imaging software. Two to 3 leaves were taken from each of 2 to 3 individual plants for **b**. One leaf was taken from each of 3 individual plants for **c**. The box plot indicates the area of trypan blue stained cells. Boxes show upper and lower quartiles of the data, and black lines represent the medians. Statistical groups were determined using the Tukey HSD test. Statistically significant differences are indicated by different letters (*p* < 0.05).
(TIF)

S16 Fig *Pto* syringe infiltration test in the *psig1* mutants. 5-week-old plants were syringe inoculated with 5 x 10^4^ c.f.u. ml^-1^ of *Pto* under long day conditions (12 h light / 12 h dark), and bacterial growth was determined at 0 and 3 dpi for **a** and at 0, 2 and 3 dpi for **b**. Data are shown as the mean ± SE. Two leaves were taken from each of 3 individual plants. Statistical groups were determined using the Tukey HSD test. Statistically significant differences are indicated by different letters (*p* < 0.01).
(TIF)

S17 Fig *Pto AvrRPS4* infiltration test in the *psig1* mutants. Plants were syringe inoculated with 5 x 10^5^ c.f.u. ml^-1^ of *Pto AvrRPS4*, and bacterial growth was determined at 3 dpi. Data are shown as the mean ± SE. Statistical groups were determined using the Tukey HSD test. Statistically significant differences are indicated by different letters (*p* < 0.05).
(TIF)

S18 Fig *PSIG1* gene expression in the WT Col-8 plants. **a**, *PSIG1* gene expression upon in leaves of soil grown plants. Six-week-old plants were syringe infiltrated with 1 x 10^6^ c.f.u. ml^-1^ of *Pto*. **b**, *PSIG1* gene expression in 10-day-old liquid culture grown seedlings. The data are shown as mean ± SE. No significant differences were observed between non-treated and treated conditions according to the two-tailed t-test (p < 0.05).
(TIF)

S19 Fig MSMS spectrum for the phosphopeptide 'DIQGSDNAIPLpSPQWLLSKPGENK'. (PDF)

S1 Table Protein Identity of PSIG homologs. (TIF)

S2 Table Protein similarity of PSIG homologs. (TIF)

S3 Table PCR primers used in this study. (PDF)

S4 Table Selected phosphoproteomics data. (XLSX)

S1 Information R script of statistical analysis of quantitative PCR data using R. (PDF)

S2 Information R script of Tukey HSD test and visualization of box plot using ggplot2. (PDF)
