# Supplementary figures and images for: The GYF domain protein PSIG1 dampens the induction of cell death during plant-pathogen interactions
Source: PLoS Genet. 2017 Oct 26;13(10):e1007037. doi: 10.1371/journal.pgen.1007037 (PMC5657617; doi:10.1371/journal.pgen.1007037)

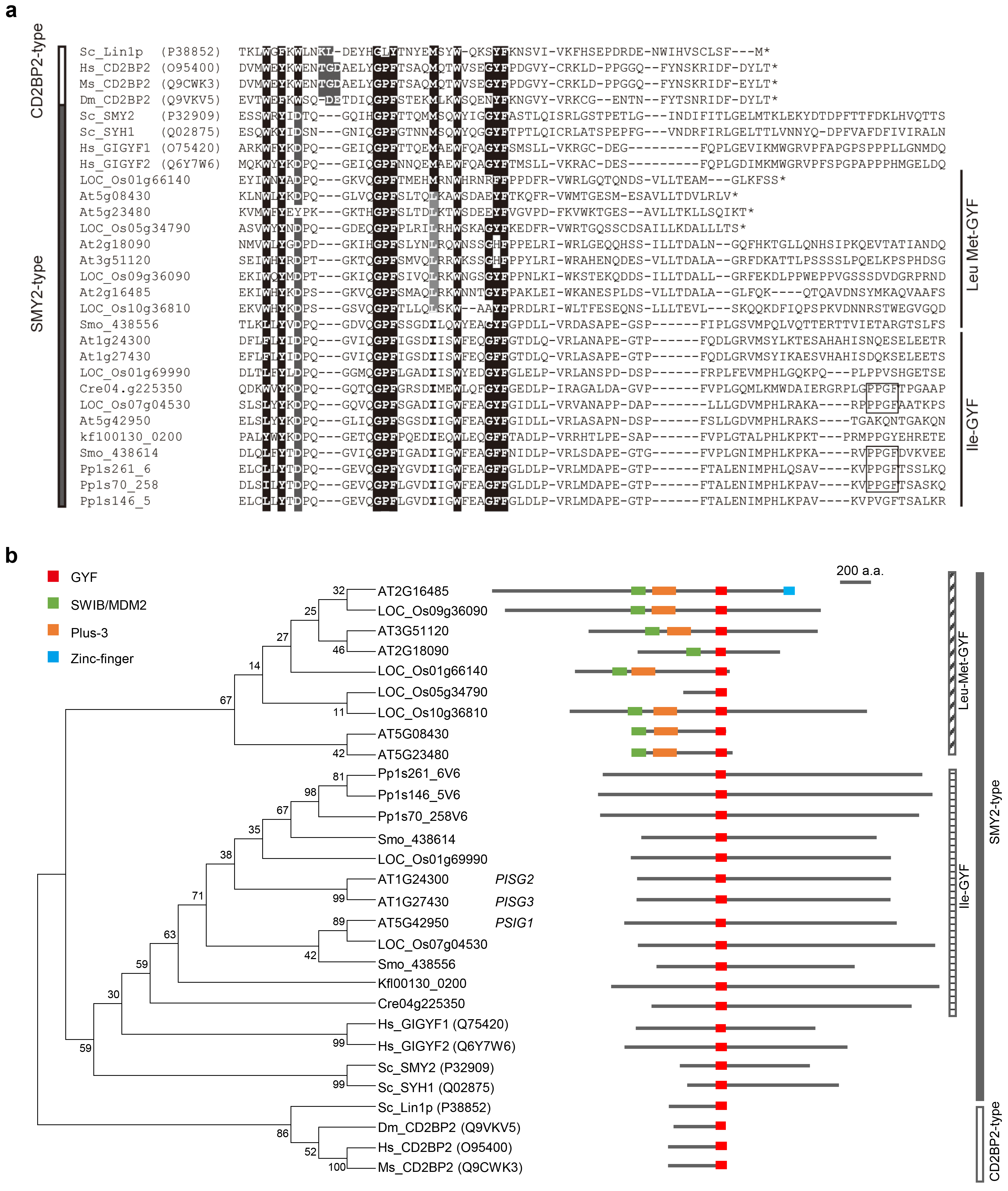

Supplement: S1 Fig — a, Aligned amino acid sequences of GYF domains from diverse eukaryotic organisms. Key residues for GYF domains are delineated as white text on a black or gray background. The conserved Ile residue of Ile-type GYF domains are indicated in bold text. At, Os, Smo, Phpat, Cre, Kfl, Hs, and Sc stand for following species: Arabidopsis thaliana, Oryza sativa, Selaginella moellendorffii, Physcomitrella patens, Chlamydomonas reinhardtii, Klebsormidium flaccidum, Homo sapiens, and Saccharomyces cerevisiae, respectively. b, Phylogenetic tree and schematic structure of GYF-domain proteins from diverse eukaryotic species. Species abbreviations are defined in S1A Fig. Numbers on the phylogenetic tree indicate the bootstrap values. Red boxes indicate the GYF domain. Green, orange and blue boxes indicate the SWIB/MDM2 domain, the Plus-3 domain and a zinc-finger domain, respectively. (TIF) [file pgen.1007037.s001.tif]

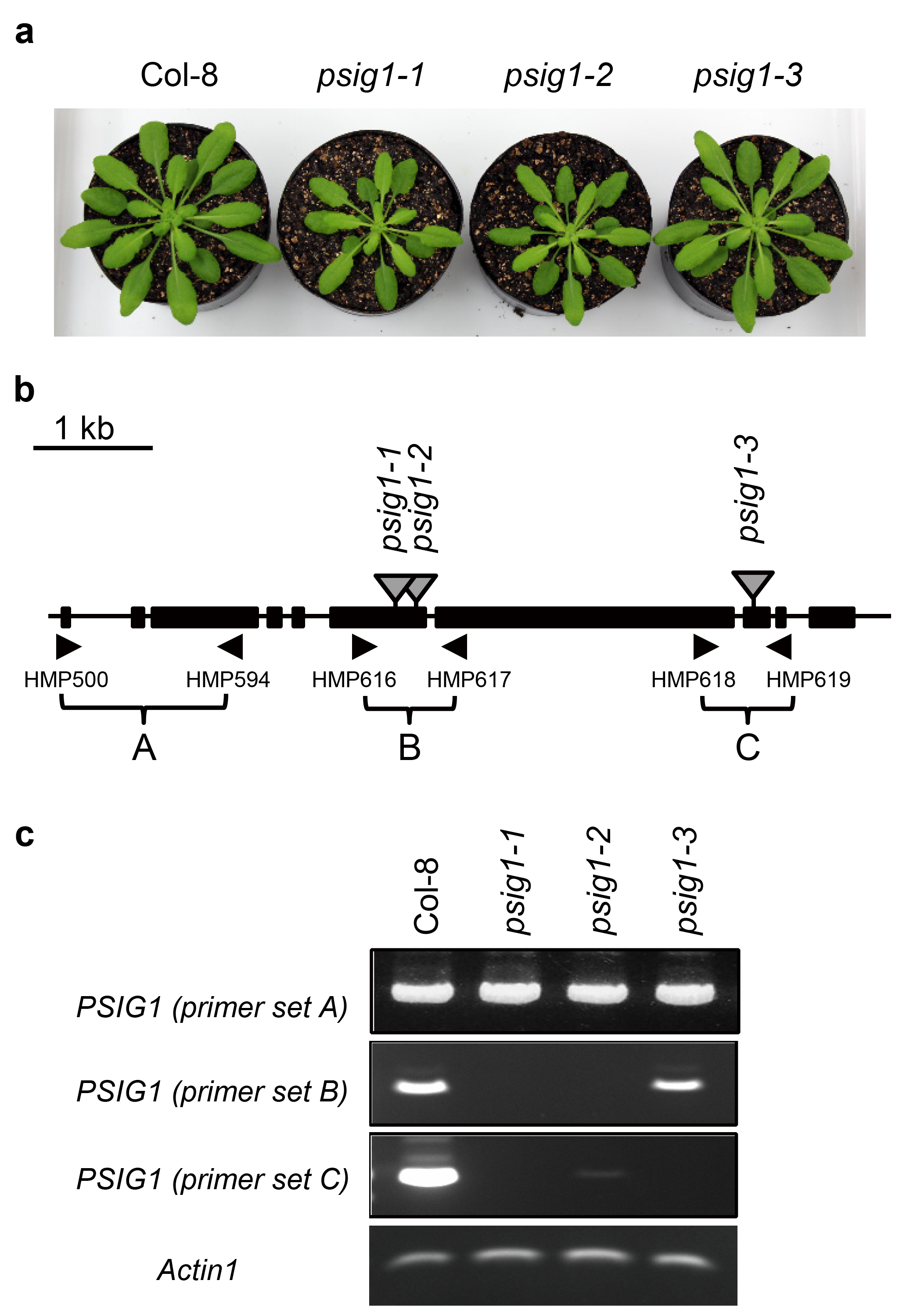

Supplement: S2 Fig — a, The psig1 mutant alleles display a slight dwarf phenotype. Photograph of 6-week-old plants grown under short day conditions. b, Genomic structure of the PSIG1 gene and the position of the T-DNA insert. Exons are indicated as black boxes. The T-DNA insertion sites are indicated by grey triangles. Black arrowheads indicate the gene-specific primer sets used for PSIG1 gene expression analysis. c, PISG1 gene expression in the psig1 mutants. Semi-quantitative RT-PCR was performed using specific primer sets as indicated in b. The Actin1 gene was used as an internal control. (TIF) [file pgen.1007037.s002.tif]

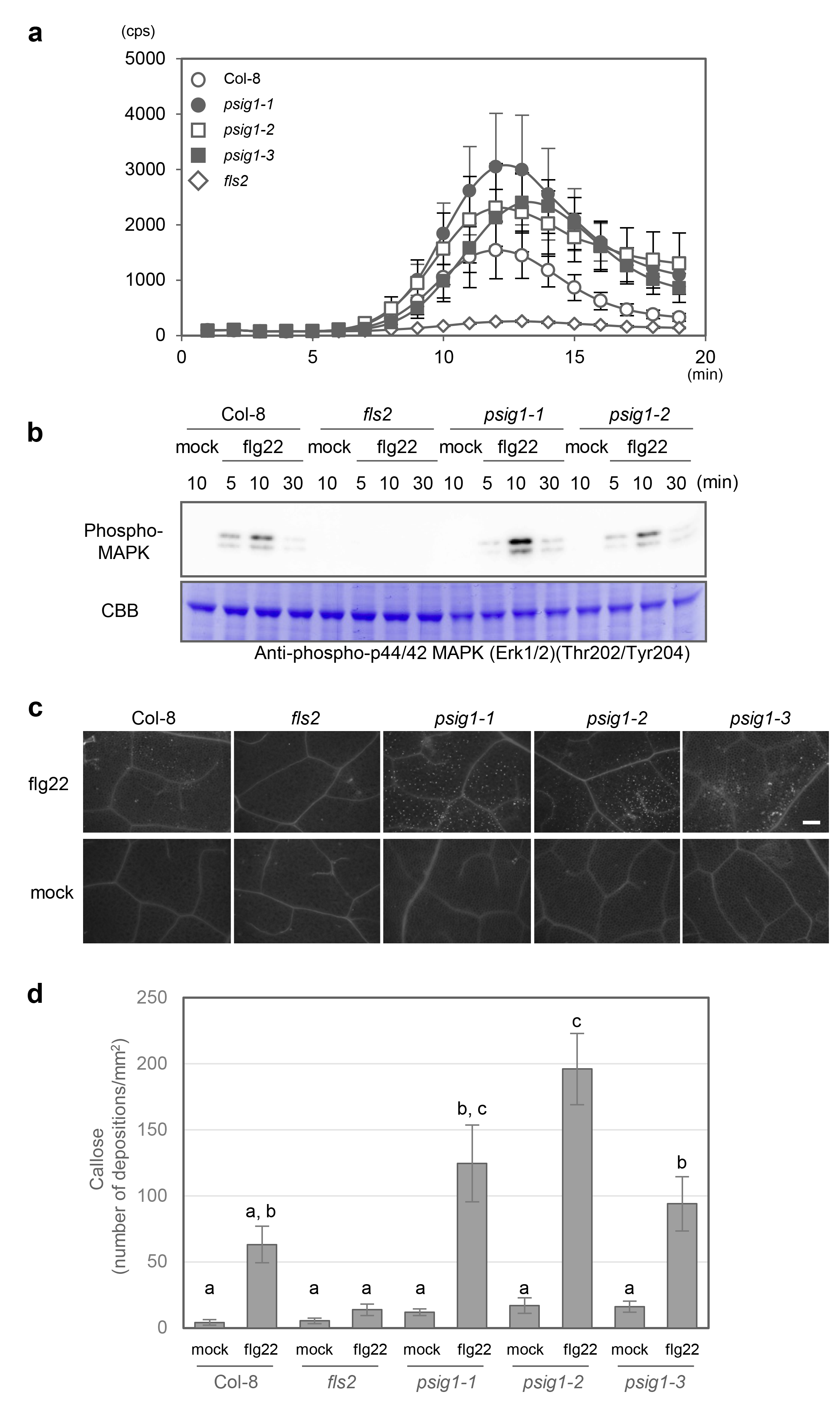

Supplement: S3 Fig — a, Flg22-induced ROS production in the psig1 mutants. Data are shown as the mean ± SE. b, Flg22-induced MAPK activation in the psig1 mutants. c, Flg22-induced callose deposition in the psig1 mutants. The scale bar represents 200 μm. d, Callose deposition was quantified with Image J software. Data are shown as the mean ± SE. Statistical groups were determined using the Tukey HSD test. Statistically significant differences are indicated by different letters (p < 0.05). (TIF) [file pgen.1007037.s003.tif]

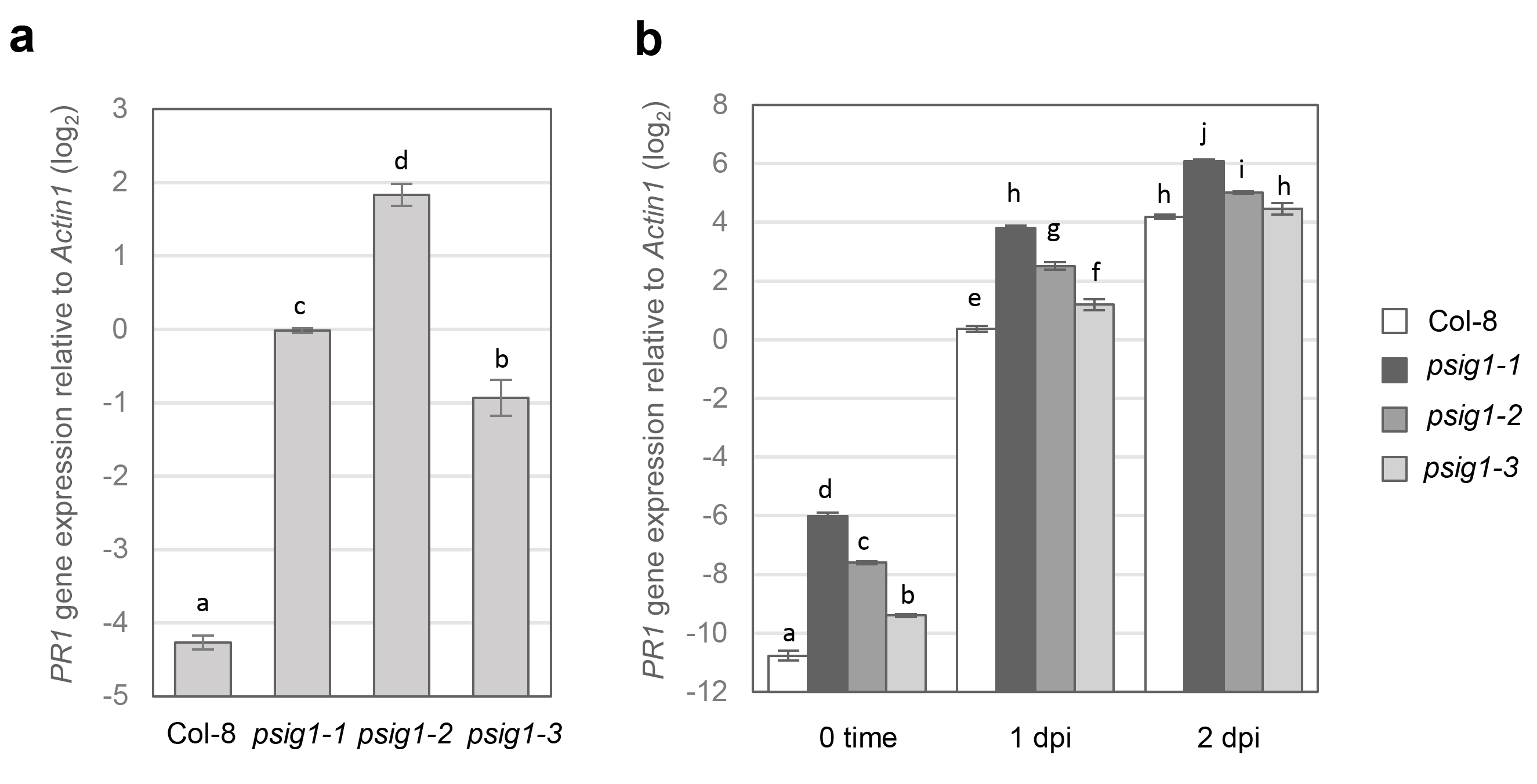

Supplement: S4 Fig — a, PR1 gene expression in 10-day-old liquid culture grown seedlings. Expression data for PR1 are shown as the mean ± SE. Statistical groups were determined using the Tukey HSD test. Statistically significant differences are indicated by different letters (p < 0.05). b, PR1 gene expression in leaves of soil grown plants. Expression data for PR1 gene are shown as the mean ± SE. Six-week-old plants were syringe infiltrated with 1 x 10^6^ c.f.u. of Pto. Statistical groups were determined using the Tukey HSD test. Statistically significant differences are indicated by different letters (p < 0.05). (TIF) [file pgen.1007037.s004.tif]

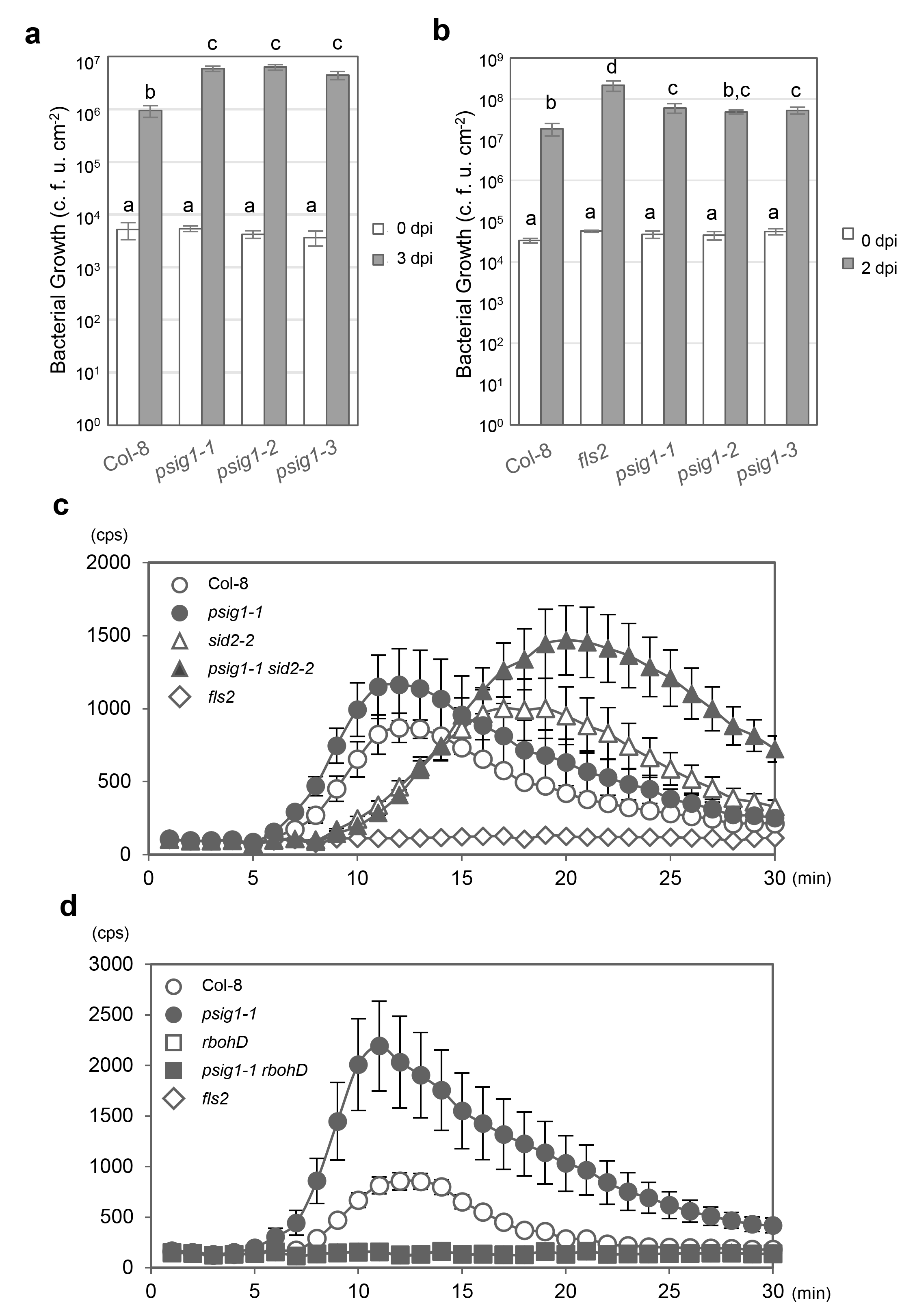

Supplement: S5 Fig — a and b, The psig1 mutants were more susceptible to Pto. Plants were spray inoculated with 1 x 10^8^ c.f.u. ml-1 of Pto, and bacterial growth was determined at 0 and 2 or 3 dpi. Data are shown as the mean ± SE. Statistical groups were determined using the Tukey HSD test. Statistically significant differences are indicated by different letters (p < 0.05). c and d, Flg22-induced ROS production in the psig1-1 sid2-2 and psig1-1 rbohD mutants. These graphs show the result of ROS production by 100 nM flg22 treatment in Arabidopsis seedlings in these genetic backgrounds. (TIF) [file pgen.1007037.s005.tif]

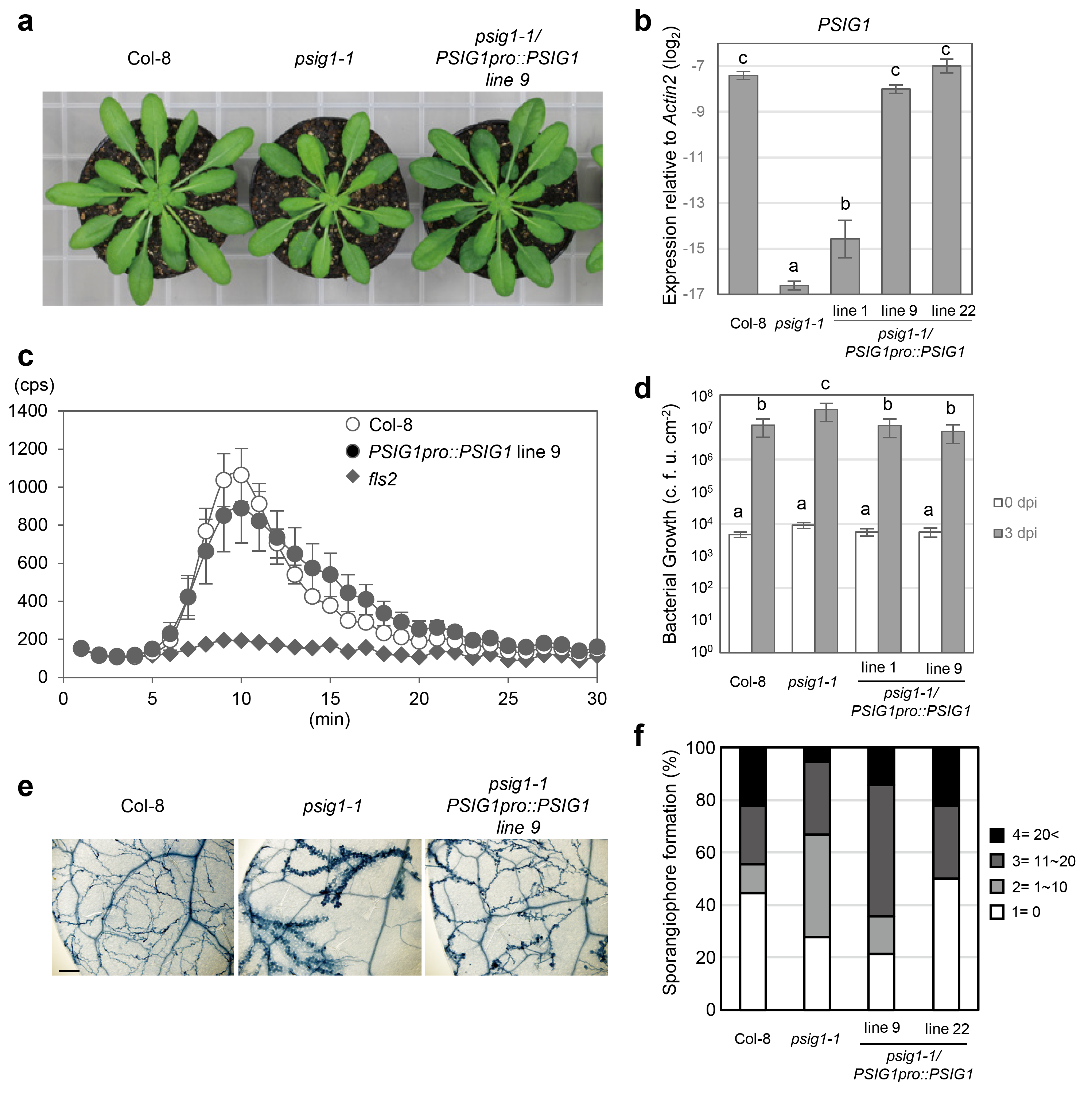

Supplement: S6 Fig — a, Photograph of 7 week-old plants grown under short day conditions. b, PSIG1 gene expression in 6-week-old plants. Data are shown as the mean ± SE. Statistical groups were determined using the Tukey HSD test. Statistically significant differences are indicated by different letters (p < 0.05). c, Flg22-induced ROS production. Data are shown as the mean ± SE. d, Pto infection. Plants were spray inoculated with 1 x 10^8^ c.f.u. ml-1 of Pto, and bacterial growth was determined at 0 and 3 dpi. Data are shown as the mean ± SE. Statistical groups were determined using the Tukey HSD test. Statistically significant differences are indicated by different letters (p < 0.05). e, Photograph of Hpa Noco2-infected leaves. Plants were inoculated with Hpa Noco2, and the true leaves were stained with trypan blue 6 days after inoculation. The scale bar represents 200 μm. f, Hpa Noco2 infection. Fourteen-day-old seedlings were inoculated with spores of Hpa Noco2, and the number of sporangiophores was scored (0 = 1, 1–10 = 2, 11–20 = 3, >20 = 4) on true leaves 6 days after inoculation. Bars show the percentage of leaves for each score (n>25). (TIF) [file pgen.1007037.s006.tif]

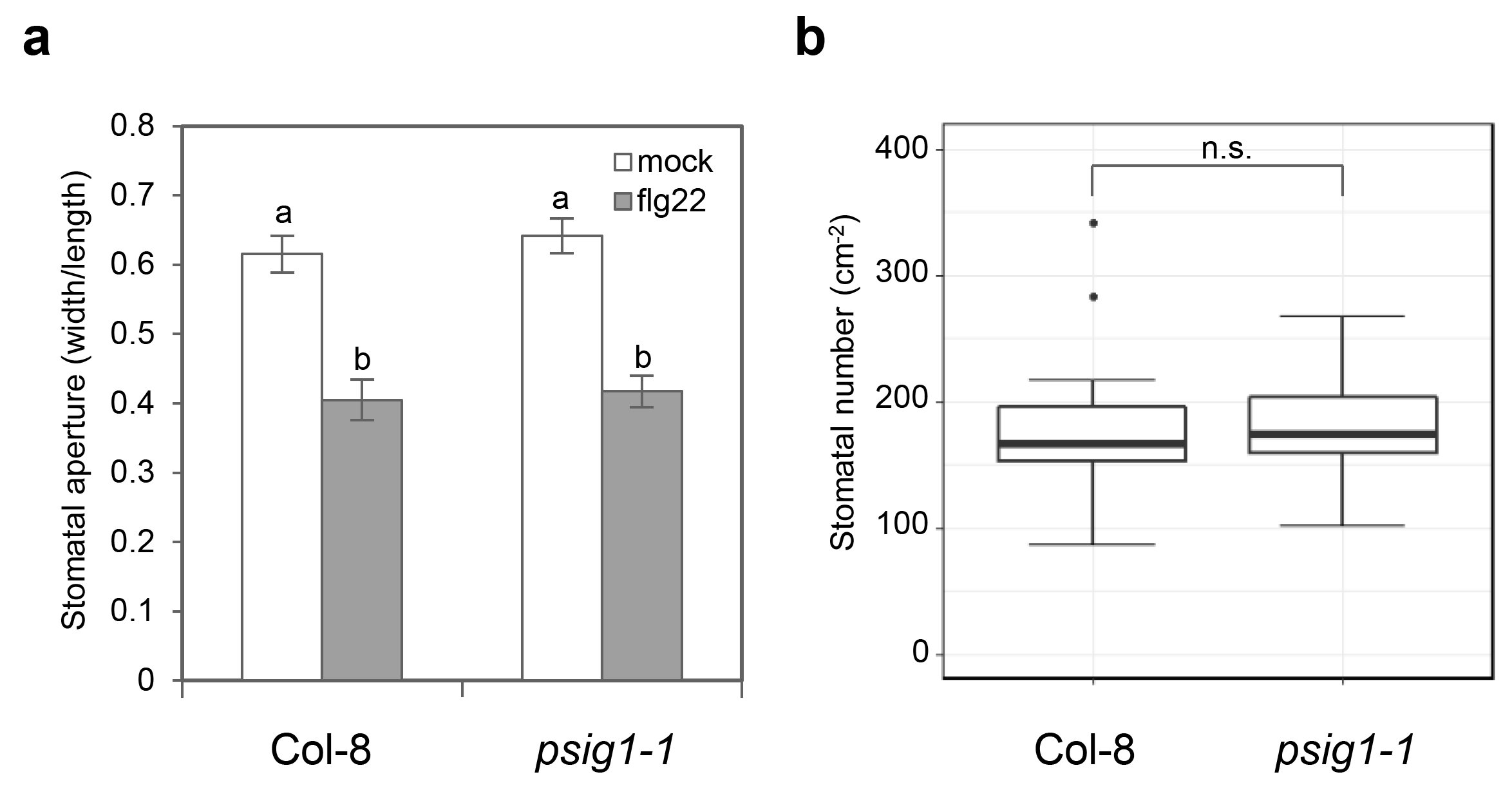

Supplement: S7 Fig — a, Flg22-induced stomatal closure in the psig1-1 mutant. Stomatal apertures were measured following treatment with 5 μM flg22 or mock (dH2O) for 60 min. Data are shown as the mean ± SE (n = 24). Statistical groups were determined using the Tukey HSD test. Statistically significant differences are indicated by different letters (p < 0.05). b, Stomatal density. Boxplots represent stomatal density (n = 36). Boxes show upper and lower quartiles of the data, and black lines represent the medians. No significant difference was observed between psig1-1 and Col-8 according to the Student t-test (p < 0.05). (TIF) [file pgen.1007037.s007.tif]

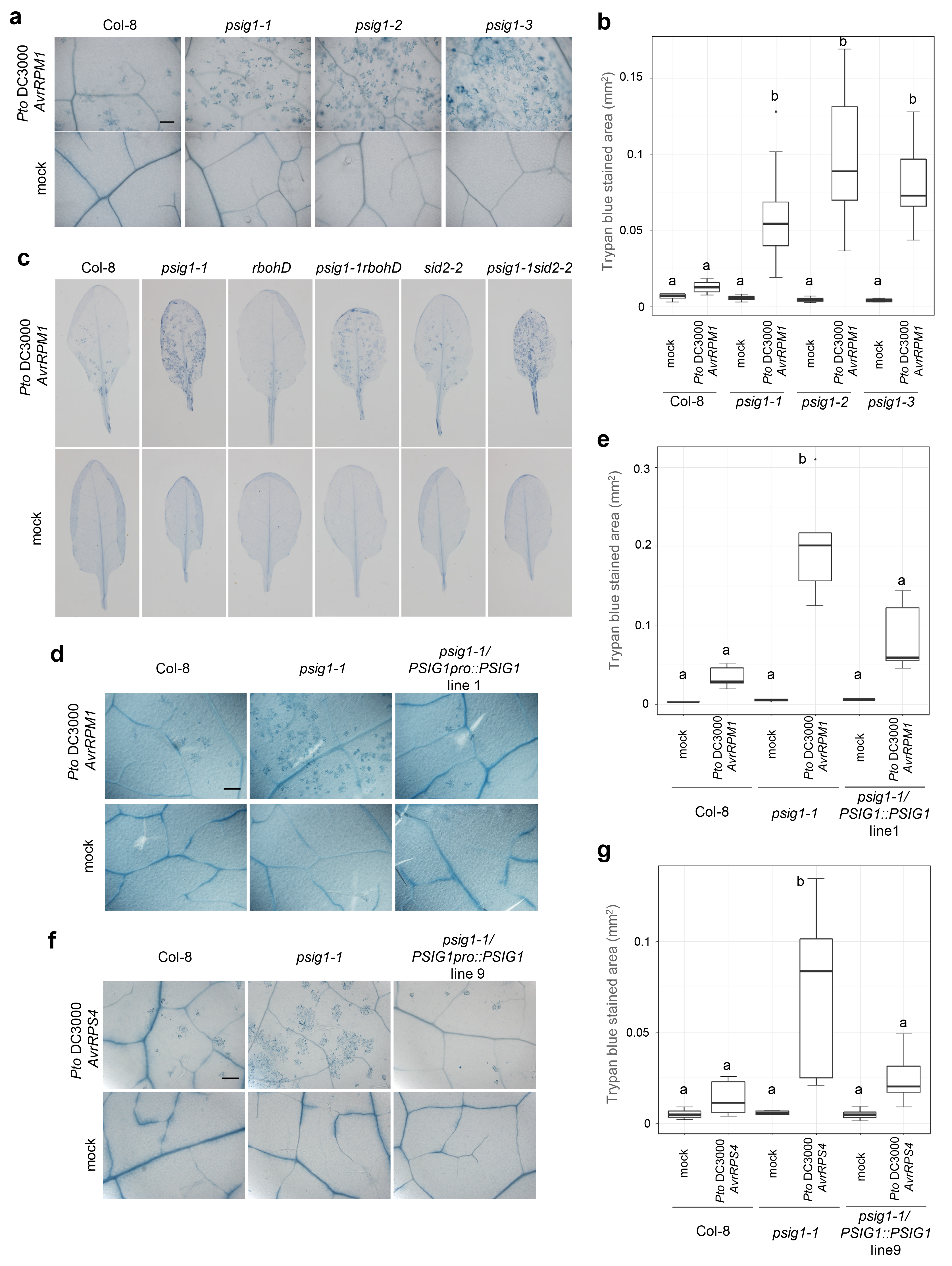

Supplement: S8 Fig — a, c, d and f, Plants were spray inoculated with 1 x 10^8^ c.f.u. ml-1 of Pto AvrRPM1 or Pto AvrRPS4, and dead cells were visualized by trypan blue staining 1 or 2 day after inoculation. The scale bar represents 200 μm. b, e and g, Trypan blue stained area. Plants were spray inoculated with 1 x 10^8^ c.f.u. ml-1 of Pto AvrRPM1 or Pto AvrRPS4, and dead cells were visualized by trypan blue staining 1 or 2 day after inoculation. The stained area was measured using an imaging software. Two leaves were taken from each of 2 individual plants for mock treatment. Three leaves were taken from each of 3 individual plants for pathogen treatment. The box plot indicates the area of trypan blue stained cells. Boxes show upper and lower quartiles of the data, and black lines represent the medians. Statistical groups were determined using the Tukey HSD test. Statistically significant differences are indicated by different letters (p < 0.05). (TIF) [file pgen.1007037.s008.tif]

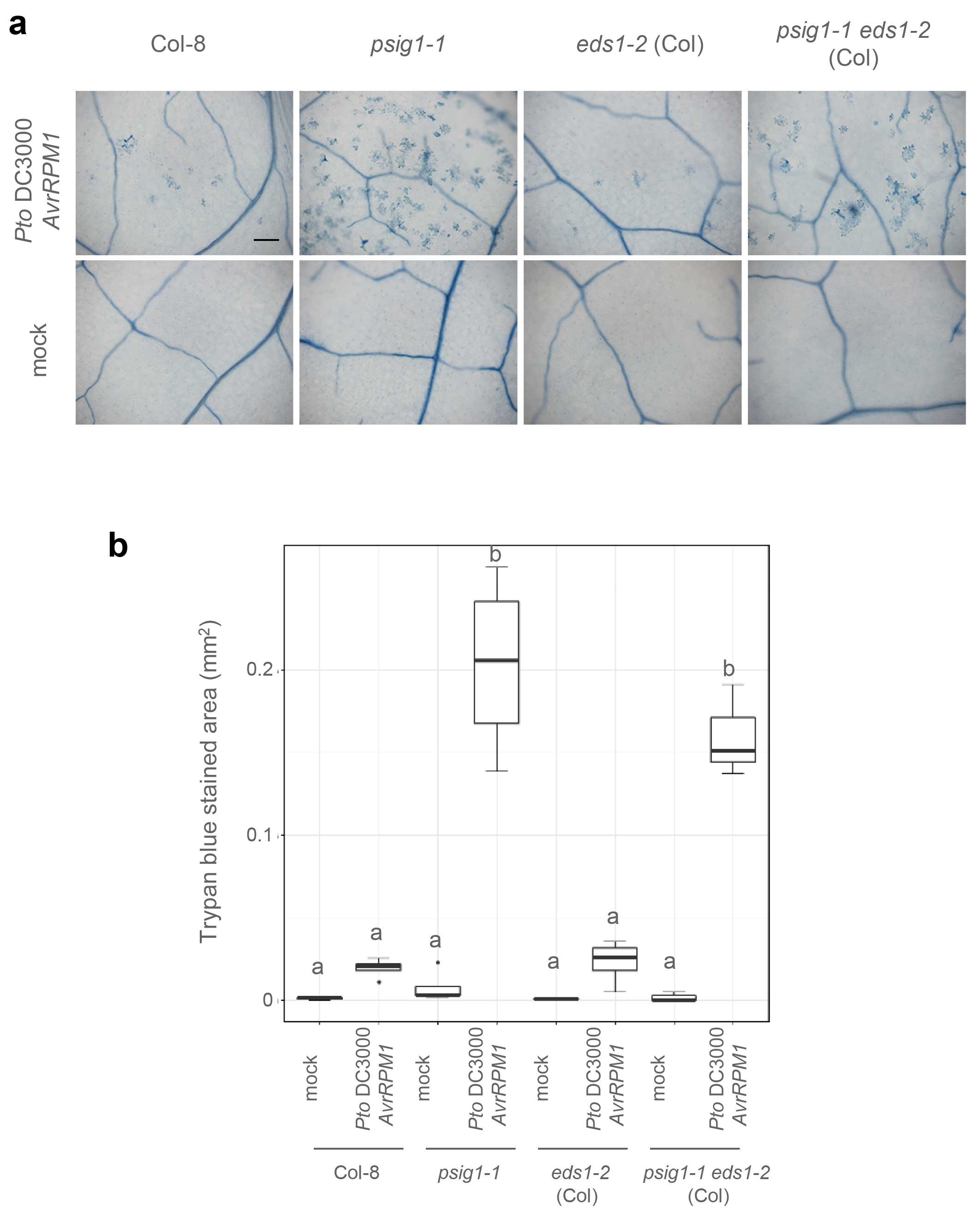

Supplement: S9 Fig — a, Plants were dip inoculated with 1 x 10^8^ c.f.u. ml-1 of Pto AvrRPM1, and dead cells were visualized by trypan blue staining 1 day after inoculation. The scale bar represents 200 μm. b, Trypan blue stained area. Plants were spray inoculated with 1 x 10^8^ c.f.u. ml-1 of Pto AvrRPM1, and dead cells were visualized by trypan blue staining 1 day after inoculation. The stained area was measured using an imaging software. Two leaves were taken from each of 2 individual plants. The box plot indicates the area of trypan blue stained cells. Boxes show upper and lower quartiles of the data, and black lines represent the medians. Statistical groups were determined using the Tukey HSD test. Statistically significant differences are indicated by different letters (p < 0.05). (TIF) [file pgen.1007037.s009.tif]

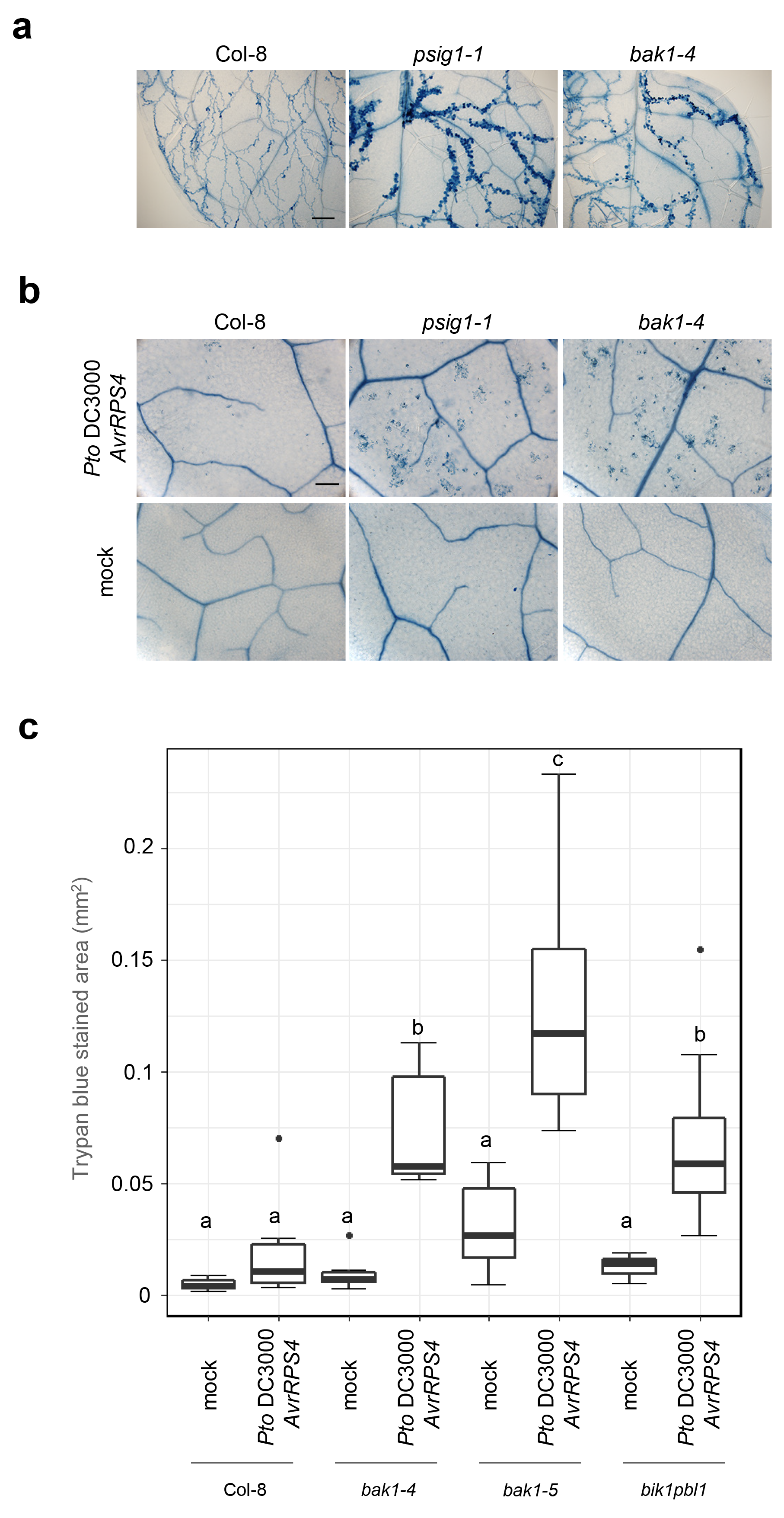

Supplement: S10 Fig — a, Plants were inoculated with Hpa Noco2 spores, and dead cells on the true leaves were visualized by trypan blue staining 6 days after inoculation. The scale bar represents 200 μm. b, Plants were spray inoculated with 1 x 10^8^ c.f.u. ml-1 of Pto AvrRPS4, and dead cells were visualized by trypan blue staining 2 days after inoculation. The scale bar represents 200 μm. c, Trypan blue stained area. Plants were spray inoculated with 1 x 10^8^ c.f.u. ml-1 of Pto AvrRPS4, and dead cells were visualized by trypan blue staining 2 days after inoculation. The stained area was measured using an imaging software. Two to 3 leaves were taken from each of 3 individual plants. The box plot indicates the area of trypan blue stained cells. Boxes show upper and lower quartiles of the data, and black lines represent the medians. Statistical groups were determined using the Tukey HSD test. Statistically significant differences are indicated by different letters (p < 0.05). (TIF) [file pgen.1007037.s010.tif]

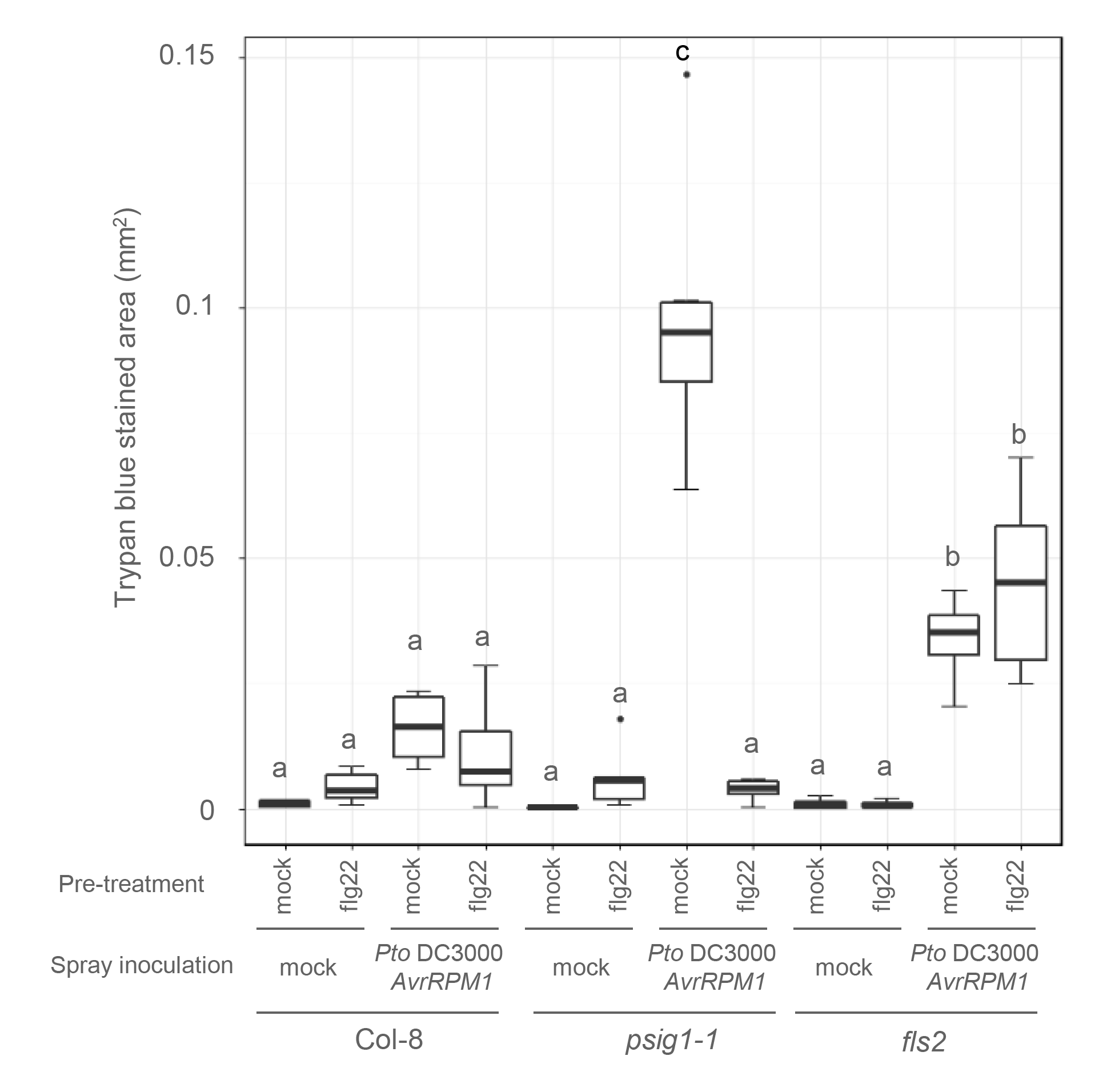

Supplement: S11 Fig — Trypan blue stained area. Pretreated plants either with 1 μM flg22 or dH2O (mock) were spray inoculated with 1 x 10^8^ c.f.u. ml-1 of Pto AvrRPM1, and dead cells were visualized by trypan blue staining 1 day after inoculation. The stained area was measured using an imaging software. Three leaves were taken from each of 3 individual plants. The box plot indicates the area of trypan blue stained cells. Boxes show upper and lower quartiles of the data, and black lines represent the medians. Statistical groups were determined using the Tukey HSD test. Statistically significant differences are indicated by different letters (p < 0.05). (TIF) [file pgen.1007037.s011.tif]

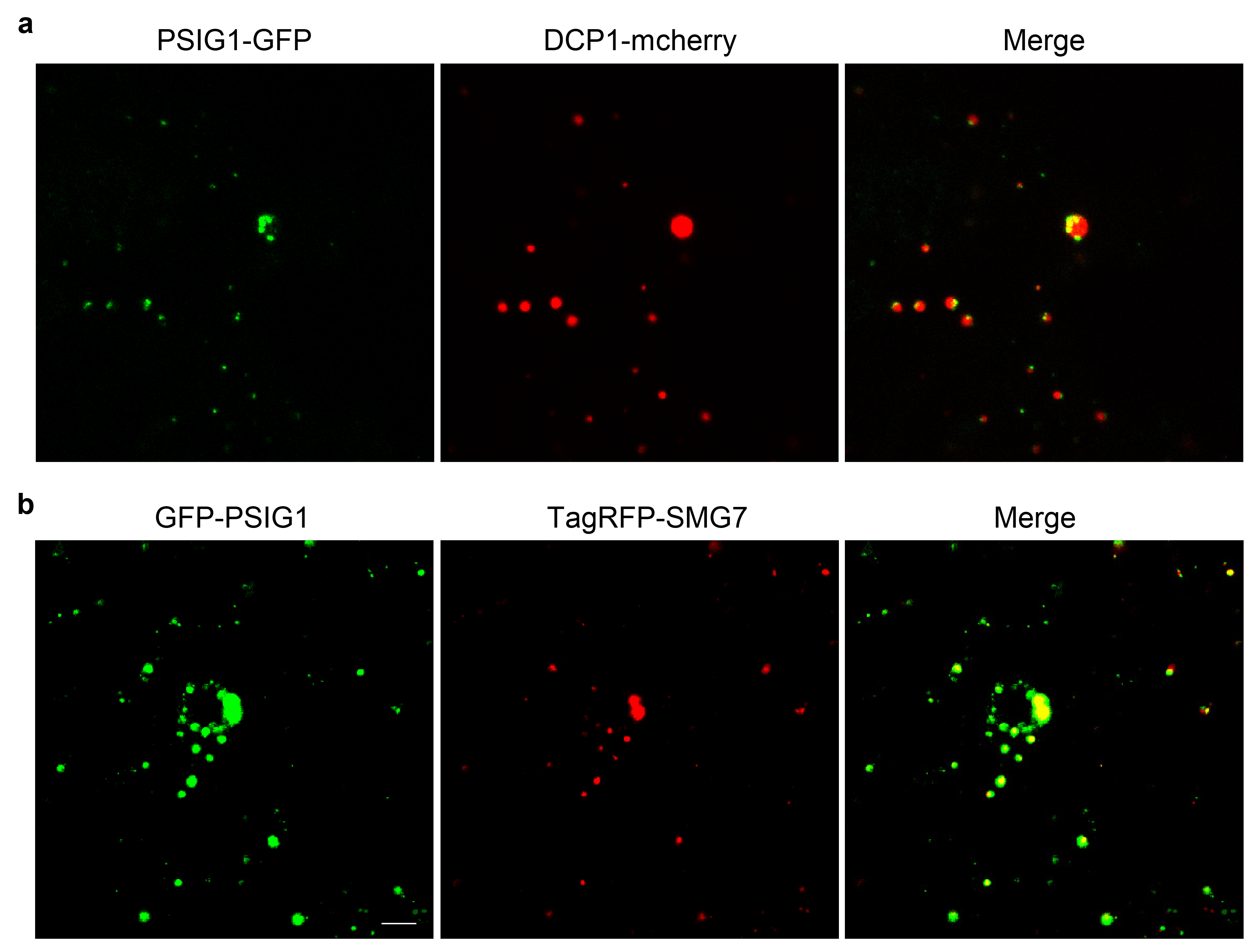

Supplement: S12 Fig — The images show the GFP signal in green and the mCherry or TagRFP signal in red. The merged images indicate the overlay of two signals in yellow. a, Subcellular localization was analyzed at 4 days after inoculation in agroinfiltrated Nicotiana benthamiana. b, Subcellular localization was analyzed at 3 days after inoculation in agroinfiltrated N. benthamiana. Before analysis, N. benthamiana was incubated at 37°C for 30 min. The scale bar represents 10 μm. (TIF) [file pgen.1007037.s012.tif]

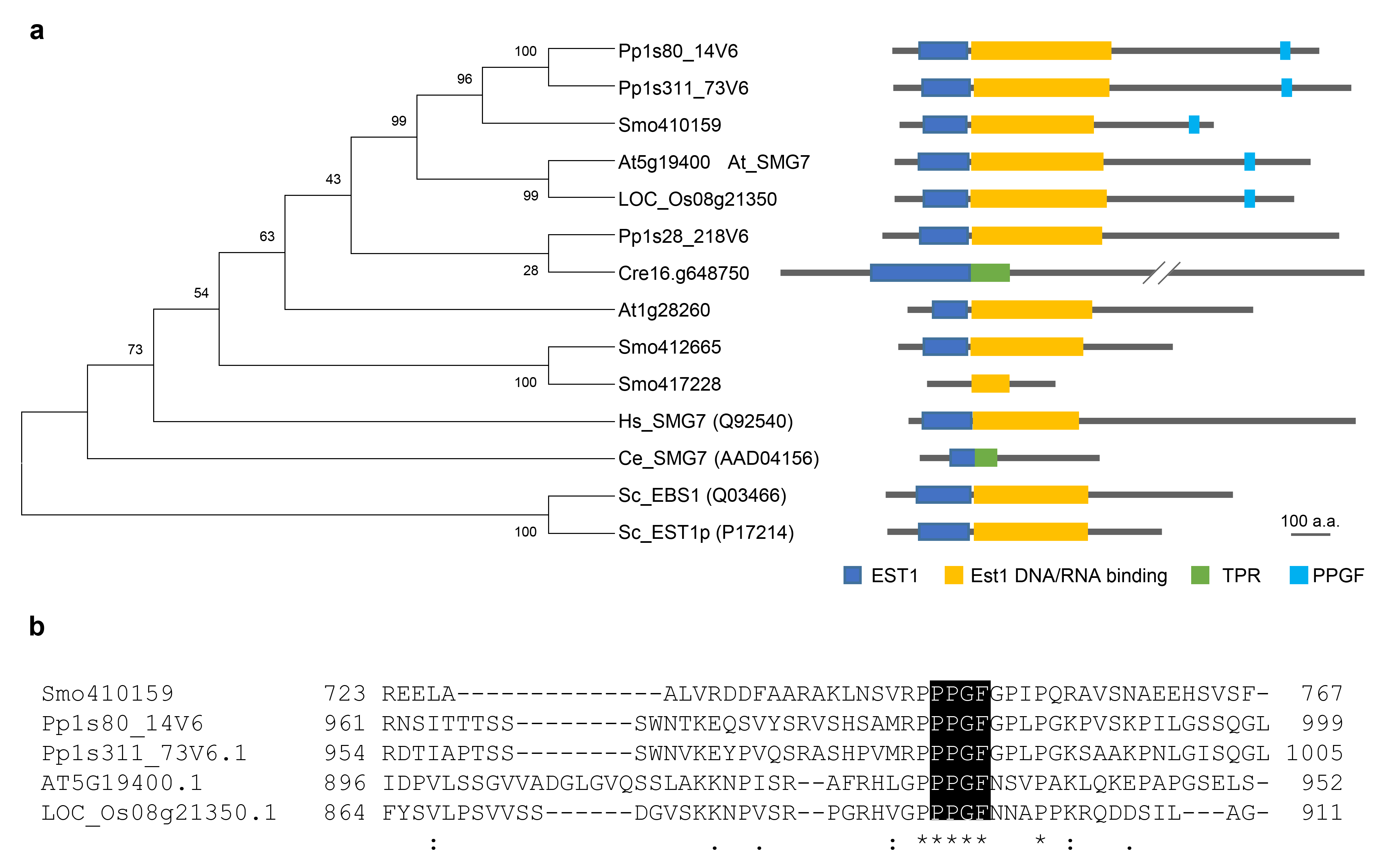

Supplement: S13 Fig — a, Phylogenetic tree and schematic structures of SMG7 from diverse eukaryotic organisms. Numbers on the phylogenetic tree indicate the bootstrap values. Dark blue boxes indicate the EST1 domain. Yellow, green and blue boxes indicate the Est1 DNA/RNA binding domain, the TPR domain and the PPGF motif, respectively. At, Os, Smo, Pp, Cre, Ce, Hs and Sc stand for following species: Arabidopsis thaliana, Oryza sativa, Selaginella moellendorffii, Physcomitrella patens, Chlamydomonas reinhardtii, Caenorhabditis elegans, Homo sapiens and Saccharomyces cerevisie, respectively. b, Aligned amino acid sequences around the PPGF sequence. The PPGF sequence is delineated as white text on a black background. (TIF) [file pgen.1007037.s013.tif]

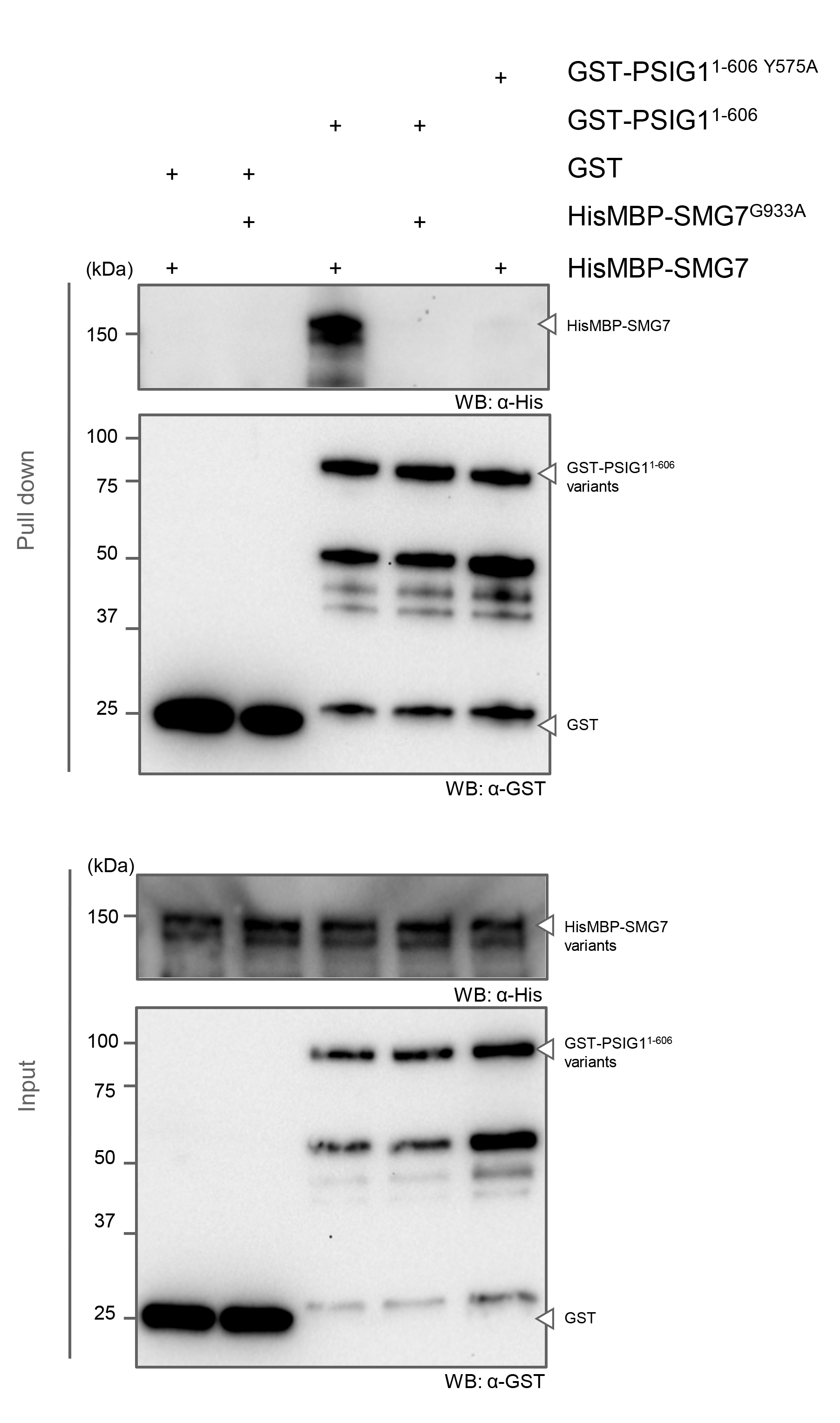

Supplement: S14 Fig — HisMBP-SMG7 or HisMBP-SMG7G933A was incubated with GST, GST-PSIG11-606, or GST-PSIG11-606 Y575A, and the conjugates were pulled down with Glutathione-Sepharose beads. HisMBP-SMG7 and GST-PSIG1 were detected by immunoblotting using anti-His antibody or anti-GST antibody. (TIF) [file pgen.1007037.s014.tif]

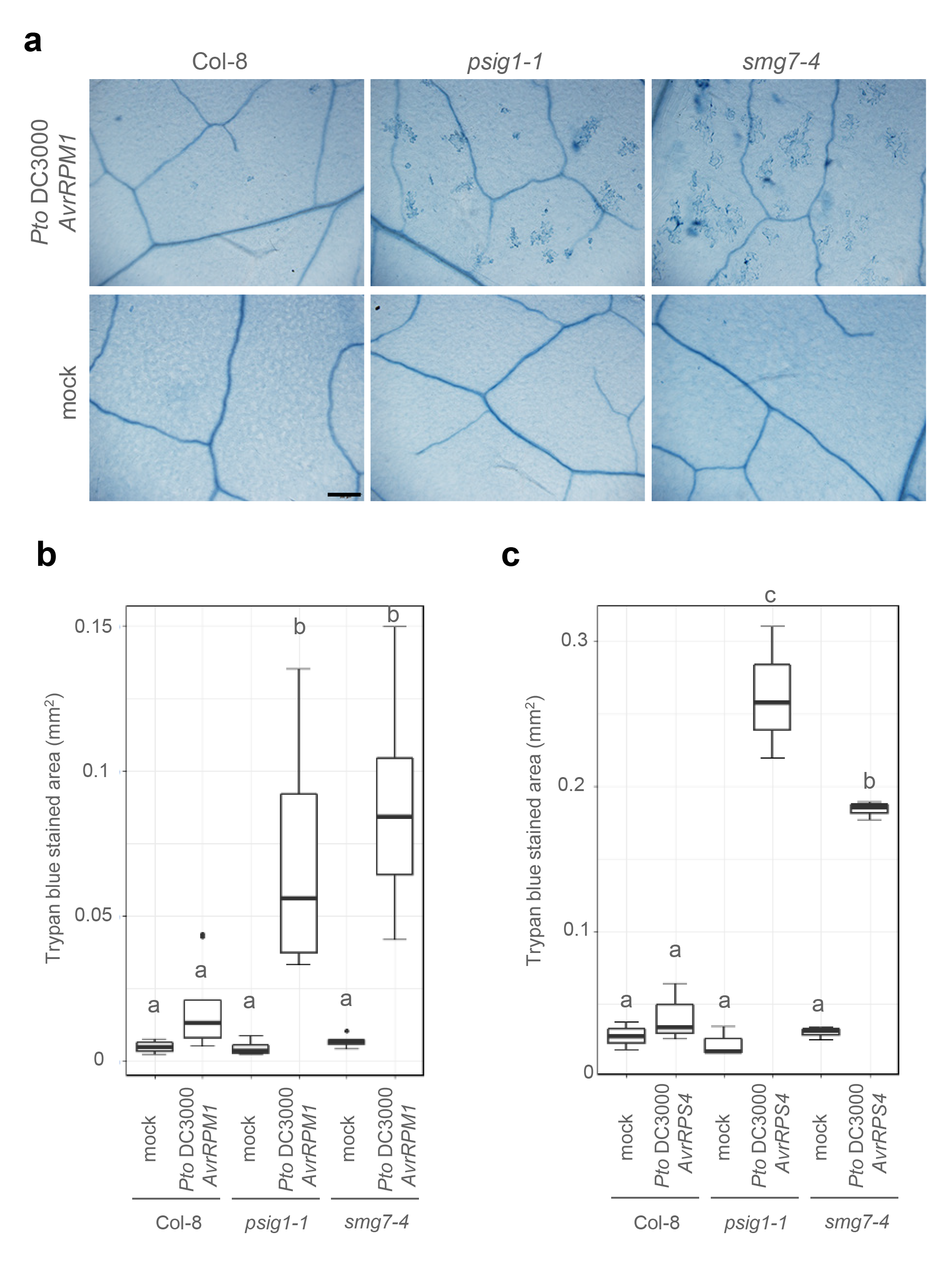

Supplement: S15 Fig — a, Plants were spray inoculated with 1 x 10^8^ c.f.u. ml-1 of Pto AvrRPM1, and dead cells were visualized by trypan blue staining 1 day after inoculation. The scale bar represents 200 μm. b and c, Trypan blue stained area. Plants were spray inoculated with 1 x 10^8^ c.f.u. ml-1 of Pto AvrRPM1 or Pto AvrRPS4, and dead cells were visualized by trypan blue staining 1 or 2 day after inoculation. The stained area was measured using an imaging software. Two to 3 leaves were taken from each of 2 to 3 individual plants for b. One leaf was taken from each of 3 individual plants for c. The box plot indicates the area of trypan blue stained cells. Boxes show upper and lower quartiles of the data, and black lines represent the medians. Statistical groups were determined using the Tukey HSD test. Statistically significant differences are indicated by different letters (p < 0.05). (TIF) [file pgen.1007037.s015.tif]

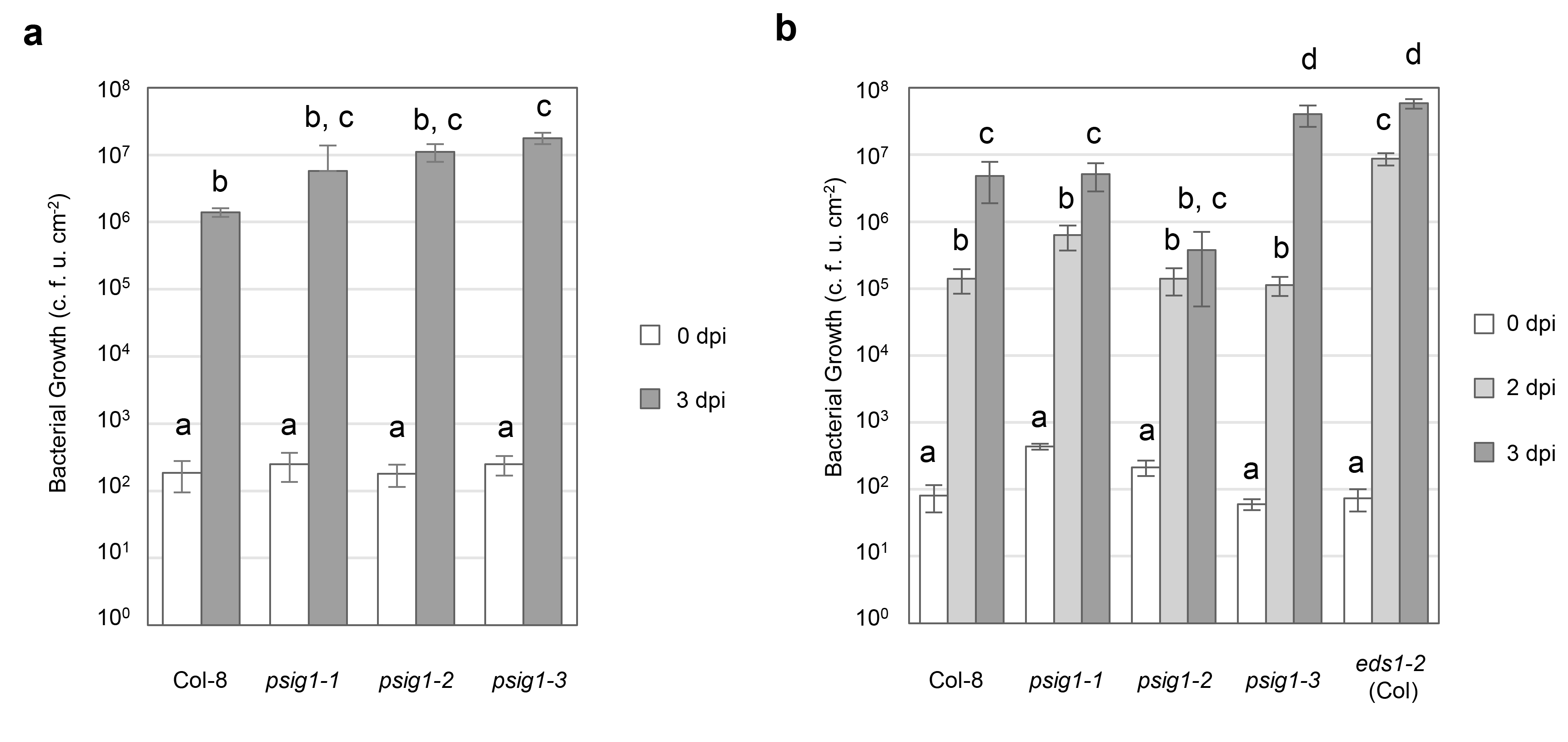

Supplement: S16 Fig — 5-week-old plants were syringe inoculated with 5 x 10^4^ c.f.u. ml-1 of Pto under long day conditions (12 h light / 12 h dark), and bacterial growth was determined at 0 and 3 dpi for a and at 0, 2 and 3 dpi for b. Data are shown as the mean ± SE. Two leaves were taken from each of 3 individual plants. Statistical groups were determined using the Tukey HSD test. Statistically significant differences are indicated by different letters (p < 0.01). (TIF) [file pgen.1007037.s016.tif]

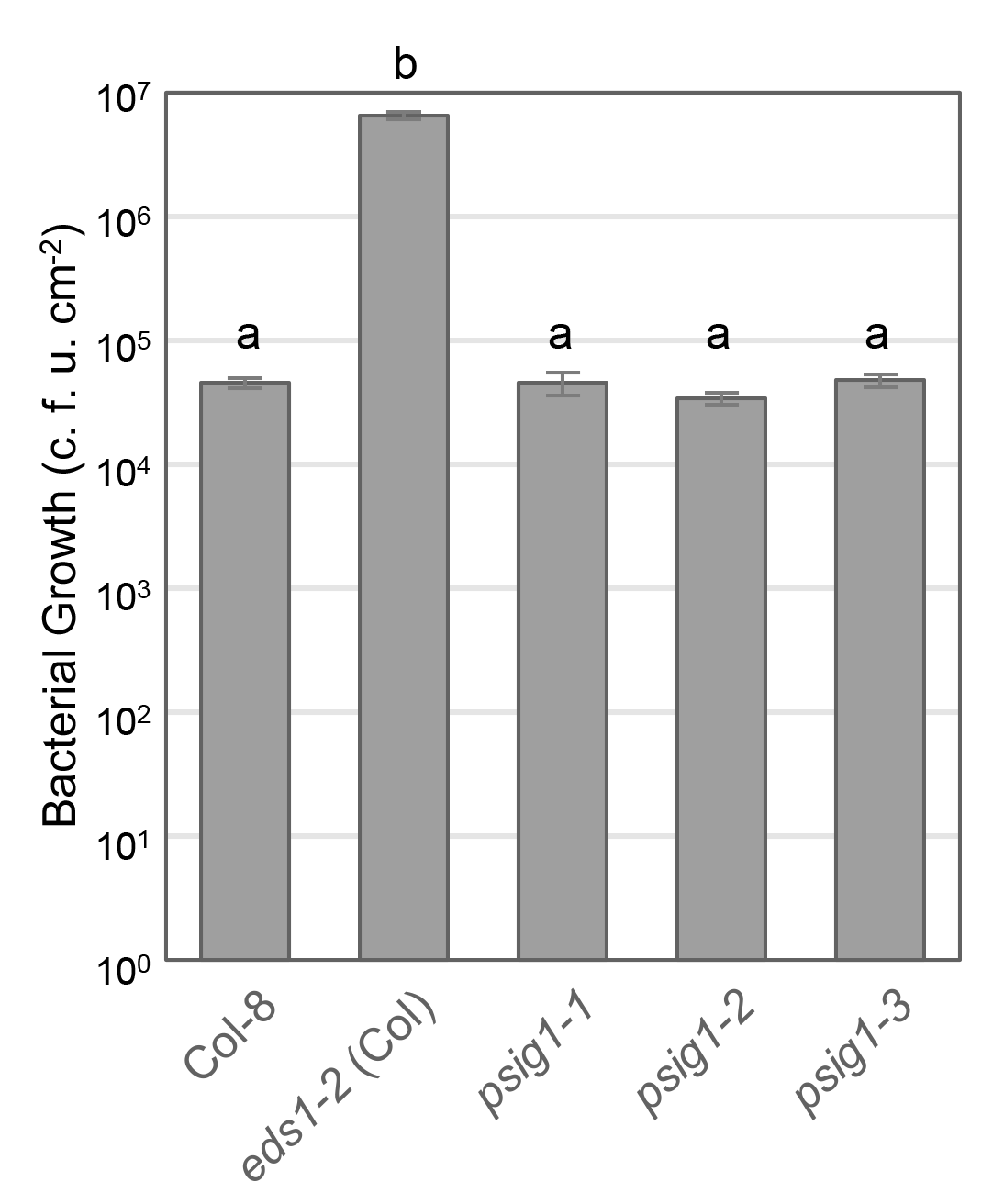

Supplement: S17 Fig — Plants were syringe inoculated with 5 x 10^5^ c.f.u. ml-1 of Pto AvrRPS4, and bacterial growth was determined at 3 dpi. Data are shown as the mean ± SE. Statistical groups were determined using the Tukey HSD test. Statistically significant differences are indicated by different letters (p < 0.05). (TIF) [file pgen.1007037.s017.tif]

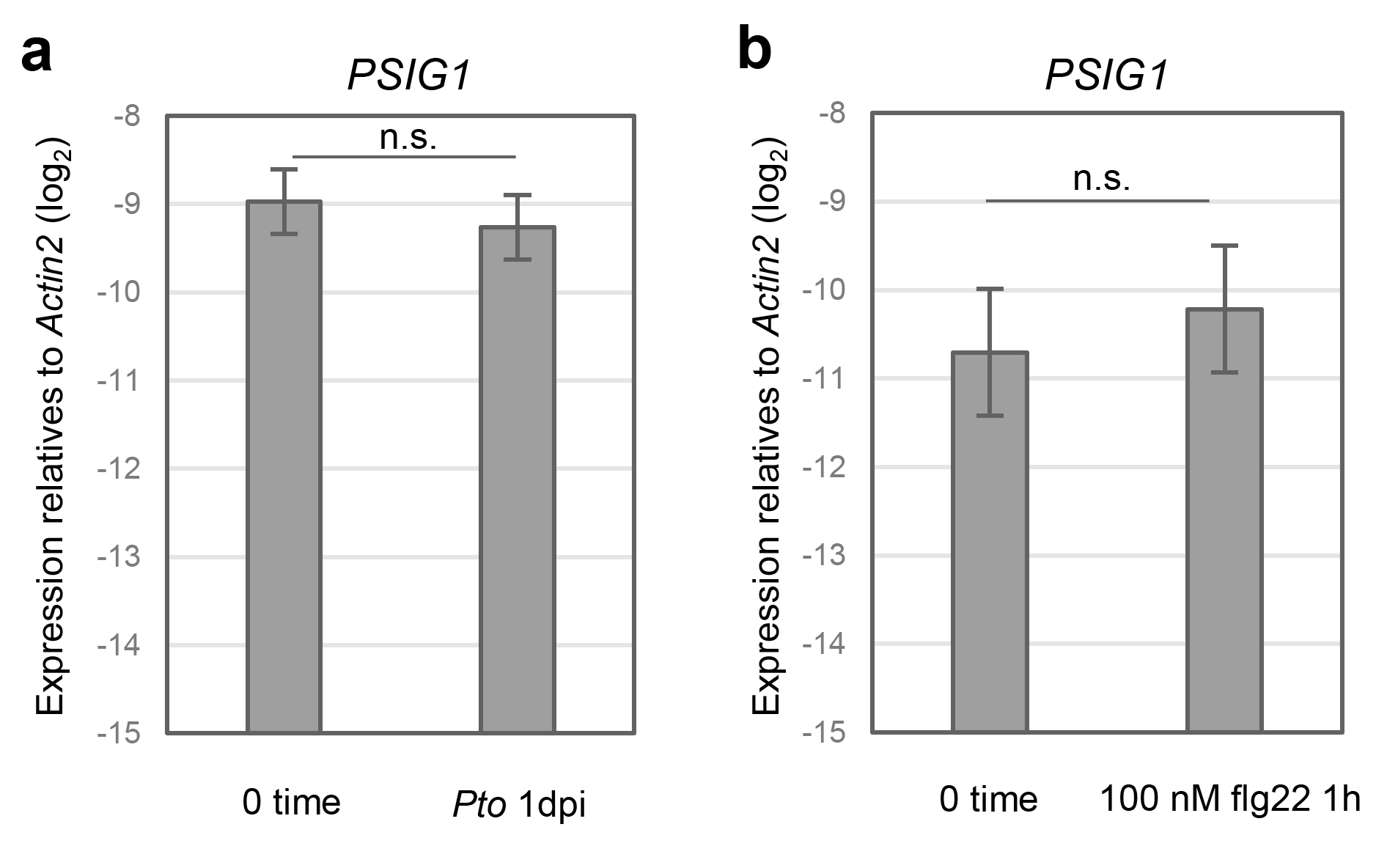

Supplement: S18 Fig — a, PSIG1 gene expression upon in leaves of soil grown plants. Six-week-old plants were syringe infiltrated with 1 x 10^6^ c.f.u. ml-1 of Pto. b, PSIG1 gene expression in 10-day-old liquid culture grown seedlings. The data are shown as mean ± SE. No significant differences were observed between non-treated and treated conditions according to the two-tailed t-test (p < 0.05). (TIF) [file pgen.1007037.s018.tif]

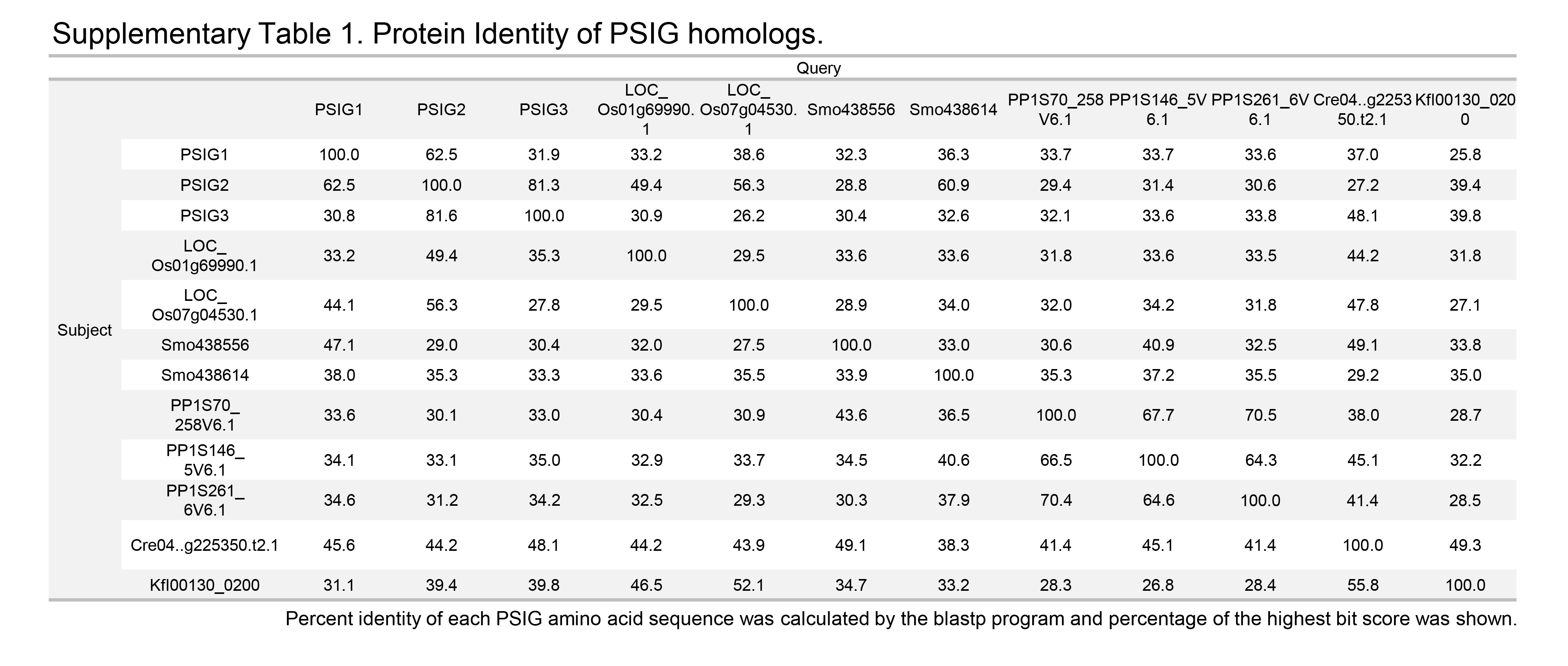

Supplement: S1 Table — (TIF) [file pgen.1007037.s020.tif]

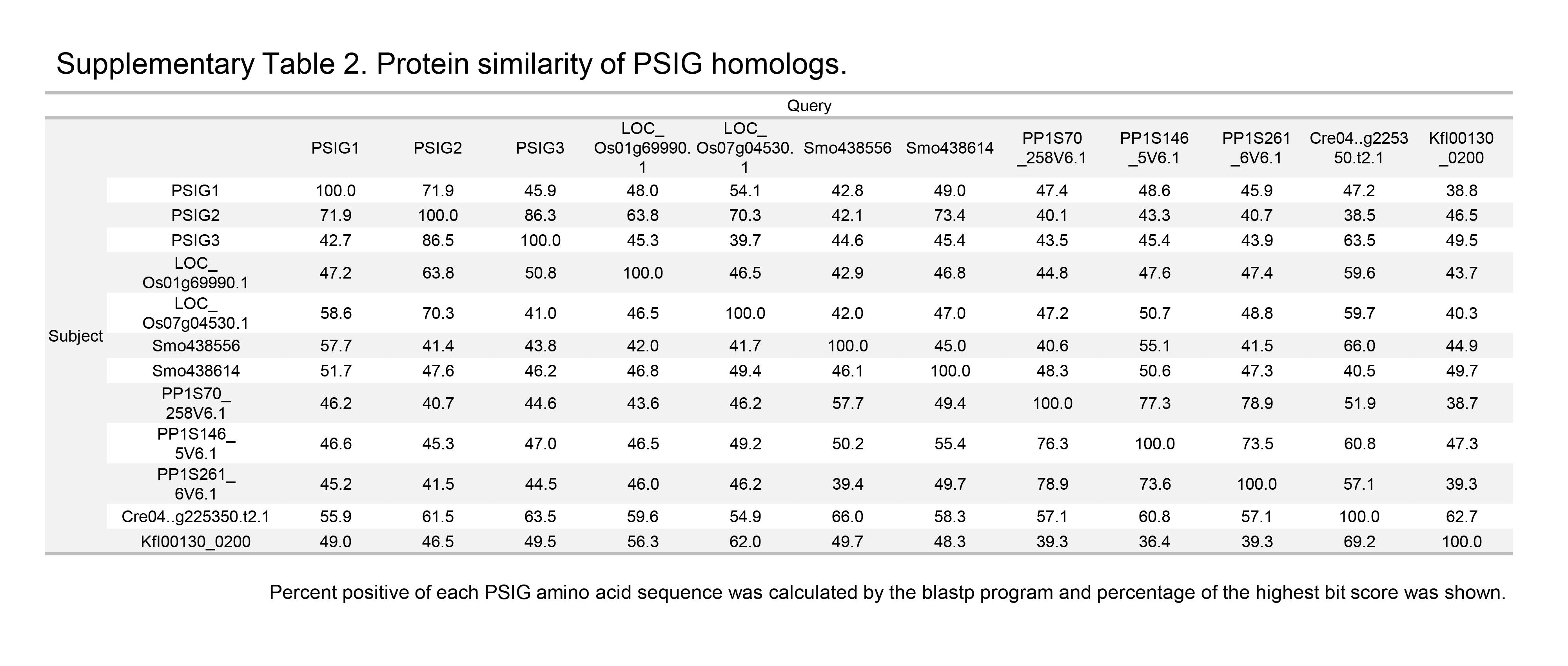

Supplement: S2 Table — (TIF) [file pgen.1007037.s021.tif]
