# Supplementary material for: The GYF domain protein PSIG1 dampens the induction of cell death during plant-pathogen interactions
Source: PLoS Genet. 2017 Oct 26;13(10):e1007037. doi: 10.1371/journal.pgen.1007037 (PMC5657617; doi:10.1371/journal.pgen.1007037)
Supplement: S19 Fig — (PDF) [file pgen.1007037.s019.pdf]

**Supplementary Figure 19. MSMS spectrum for the phosphopeptide 'DIQGSDNAIPLpSPQWLLSKPGENK'.**

# {MATRIX SCIENCE} Mascot Search Results

## Peptide View

MS/MS Fragmentation of **DIQGSDNAIPLSPQWLLSKPGENK**
Found in **AT5G42950.1** in **TAIR10_Arab**, AT5G42950.1 | Symbols: | GYF domain-containing protein | chr5:17224436-17231044 FORWARD LENGTH=1714

Match to Query 3114: 2686.303950 from(896.441926,3+) index(3184)
Title: Elution from: 81.314 to 81.314 scan no 5316 cid35.00 polarity:+
Data file 110819Orbi_13_1uM_Flg22_30min-1.txt

Click mouse within plot area to zoom in by factor of two about that point
Or, [Plot from] [400] to [1700] Da [Full range]
Label all possible matches ○ Label matches used for scoring ⦿
Show Y-axis ☐

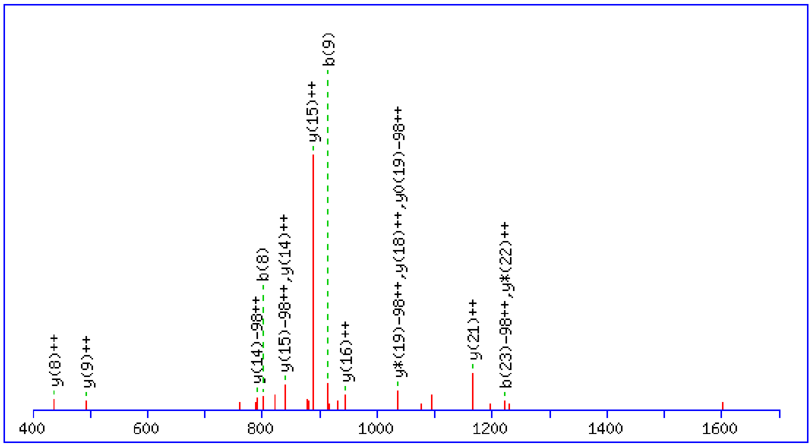


**Monoisotopic mass of neutral peptide Mr(calc):** 2686.3003
**Fixed modifications:** Carbamidomethyl (C) (apply to specified residues or termini only)
**Variable modifications:**
**S12** : Phospho (ST), with neutral losses 0.0000(shown in table), 97.9769
**Ions Score:** 41 **Expect:** 0.00084
**Matches** : 15/406 fragment ions using 16 most intense peaks (help)

| # | b | $b^{++}$ | b* | $b^{*++}$ | $b^0$ | $b^{0++}$ | Seq. | y | $y^{++}$ | y* | $y^{*++}$ | $y^0$ | $y^{0++}$ | # |
|---|---|---|---|---|---|---|---|---|---|---|---|---|---|---|
| 1 | 116.0342 | 58.5207 | | | 98.0237 | 49.5155 | D | | | | | | | 24 |
| 2 | 229.1183 | 115.0628 | | | 211.1077 | 106.0575 | I | 2572.2807 | 1286.6440 | 2555.2541 | 1278.1307 | 2554.2701 | 1277.6387 | 23 |
| 3 | 357.1769 | 179.0921 | 340.1503 | 170.5788 | 339.1663 | 170.0868 | Q | 2459.1966 | 1230.1020 | 2442.1701 | 1221.5887 | 2441.1861 | 1221.0967 | 22 |
| 4 | 414.1983 | 207.6028 | 397.1718 | 199.0895 | 396.1878 | 198.5975 | G | 2331.1381 | 1166.0727 | 2314.1115 | 1157.5594 | 2313.1275 | 1157.0674 | 21 |
| 5 | 501.2304 | 251.1188 | 484.2038 | 242.6055 | 483.2198 | 242.1135 | S | 2274.1166 | 1137.5619 | 2257.0900 | 1129.0487 | 2256.1060 | 1128.5566 | 20 |
| 6 | 616.2573 | 308.6323 | 599.2307 | 300.1190 | 598.2467 | 299.6270 | D | 2187.0846 | 1094.0459 | 2170.0580 | 1085.5326 | 2169.0740 | 1085.0406 | 19 |
| 7 | 730.3002 | 365.6537 | 713.2737 | 357.1405 | 712.2897 | 356.6485 | N | 2072.0576 | 1036.5324 | 2055.0311 | 1028.0192 | 2054.0471 | 1027.5272 | 18 |
| 8 | **801.3373** | 401.1723 | 784.3108 | 392.6590 | 783.3268 | 392.1670 | A | 1958.0147 | 979.5110 | 1940.9881 | 970.9977 | 1940.0041 | 970.5057 | 17 |
| 9 | **914.4214** | 457.7143 | 897.3949 | 449.2011 | 896.4108 | 448.7091 | I | 1886.9776 | 943.9924 | 1869.9510 | 935.4792 | 1868.9670 | 934.9871 | 16 |
| 10 | 1011.4742 | 506.2407 | 994.4476 | 497.7274 | 993.4636 | 497.2354 | P | 1773.8935 | 887.4504 | 1756.8670 | 878.9371 | 1755.8829 | 878.4451 | 15 |
| 11 | 1124.5582 | 562.7828 | 1107.5317 | 554.2695 | 1106.5477 | 553.7775 | L | 1676.8407 | 838.9240 | 1659.8142 | 830.4107 | 1658.8302 | 829.9187 | 14 |
| 12 | 1291.5566 | 646.2819 | 1274.5300 | 637.7687 | 1273.5460 | 637.2766 | S | 1563.7567 | 782.3820 | 1546.7301 | 773.8687 | 1545.7461 | 773.3767 | 13 |
| 13 | 1388.6094 | 694.8083 | 1371.5828 | 686.2950 | 1370.5988 | 685.8030 | P | 1396.7583 | 698.8828 | 1379.7318 | 690.3695 | 1378.7478 | 689.8775 | 12 |
| 14 | 1516.6679 | 758.8376 | 1499.6414 | 750.3243 | 1498.6574 | 749.8323 | Q | 1299.7056 | 650.3564 | 1282.6790 | 641.8431 | 1281.6950 | 641.3511 | 11 |
| 15 | 1702.7472 | 851.8773 | 1685.7207 | 843.3640 | 1684.7367 | 842.8720 | W | 1171.6470 | 586.3271 | 1154.6204 | 577.8139 | 1153.6364 | 577.3218 | 10 |
| 16 | 1815.8313 | 908.4193 | 1798.8048 | 899.9060 | 1797.8207 | 899.4140 | L | 985.5677 | 493.2875 | 968.5411 | 484.7742 | 967.5571 | 484.2822 | 9 |
| 17 | 1928.9154 | 964.9613 | 1911.8888 | 956.4480 | 1910.9048 | 955.9560 | L | 872.4836 | 436.7454 | 855.4571 | 428.2322 | 854.4730 | 427.7402 | 8 |
| 18 | 2015.9474 | 1008.4773 | 1998.9209 | 999.9641 | 1997.9368 | 999.4721 | S | 759.3995 | 380.2034 | 742.3730 | 371.6901 | 741.3890 | 371.1981 | 7 |
| 19 | 2144.0424 | 1072.5248 | 2127.0158 | 1064.0115 | 2126.0318 | 1063.5195 | K | 672.3675 | 336.6874 | 655.3410 | 328.1741 | 654.3569 | 327.6821 | 6 |
| 20 | 2241.0951 | 1121.0512 | 2224.0686 | 1112.5379 | 2223.0846 | 1112.0459 | P | 544.2726 | 272.6399 | 527.2460 | 264.1266 | 526.2620 | 263.6346 | 5 |
| 21 | 2298.1166 | 1149.5619 | 2281.0900 | 1141.0487 | 2280.1060 | 1140.5567 | G | 447.2198 | 224.1135 | 430.1932 | 215.6003 | 429.2092 | 215.1082 | 4 |
| 22 | 2427.1592 | 1214.0832 | 2410.1326 | 1205.5700 | 2409.1486 | 1205.0779 | E | 390.1983 | 195.6028 | 373.1718 | 187.0895 | 372.1878 | 186.5975 | 3 |
| 23 | 2541.2021 | 1271.1047 | 2524.1756 | 1262.5914 | 2523.1915 | 1262.0994 | N | 261.1557 | 131.0815 | 244.1292 | 122.5682 | | | 2 |
| 24 | | | | | | | K | 147.1128 | 74.0600 | 130.0863 | 65.5468 | | | 1 |
