# Supplementary material for: The GYF domain protein PSIG1 dampens the induction of cell death during plant-pathogen interactions
Source: PLoS Genet. 2017 Oct 26;13(10):e1007037. doi: 10.1371/journal.pgen.1007037 (PMC5657617; doi:10.1371/journal.pgen.1007037)
Supplement: S3 Table — (PDF) [file pgen.1007037.s022.pdf]

Supplementary Table 3. PCR primers used in this study.

	Name	5'-sequence-3'	
psig1-1 T-DNA genotyping	SALK_LBb1	GCGTGGACCGCTTGCTGCAACT	in this study
	psig1-1 LP	ATGCCACCTGGATTATTTTCC	
	psig1-1 RP	GAGGCACCTGTCAGAGAACTG	
psig1-2 T-DNA genotyping	*psig1-2* LP	ACCAATAGACGCTGATGTTGG	in this study
	psig1-2 RP	AGAAAGCTTCTCCCATCTTCG	
psig1-3 T-DNA genotyping	SAIL1_LB	GCCTTTTCAGAAATGGATAAATAGCCTTGCTTCC	in this study
	psig1-3 LP	AAAAATTGCTGCACATGTTCC	
	psig1-3 RP	AGAGTGCTTAGGGTAGCCACC	
sid2-2 genotyping		TTCTTCATGCAGGGGAGGAG	Dewdney et al., 2000
		CAACCACCTGGTGCACCAGC	
		AAGCAAAATGTTTGAGTCAGCA	
rbohd genotyping	dspm1	CTTATTTCAGTAAGAGTGTGGGGTTTTGG	Torres et al., 2002
	dspm11	GGTGCAGCAAAACCCACACTTTTACTTC	
	D122	ATGAAAATGAGACGAGGCAATTC	
	D92b	GGATACTGATCATAGGCGTGGCTCCA	
eds1-2 (Col) genotyping	105/E2	ACACAAGGGTGATGCGAGACA	Gloggnitzer et al., 2014
	EDS4	GGCTTGTATTCATCTTCTATCC	
	EDS6	GTGGAAACCAAATTTGACATTAG	
Quantitative RT-PCR	*Actin2* FW	ACACTGTGCCAATCTACGAGGG	Trujillo et al., 2008
	Actin2 RV	CTCTTACAATTTCCCGCTCTGC	
	Actin1 FW	TCTTGATCTTGCTGGTCGTG	
	Actin1 RV	GAGCTGGTTTTGGCTGTCTC	
	PR1 FW	TTCTTCCCTCGAAAGCTCAA	
	PR1 RV	AAGGCCCACCAGAGTGTATG	
Semi RT-PCR	*PSIG1* RT-Primer set A FW	caccATGGCTAACTCTTCCGCTGGCTCCGCCGCAGACCAC	in this study
	PSIG1 RT-Primer set A RV	CGGGCTAAGTCGGGTCCACCACCTGG	
	PSIG1 RT-Primer set B FW	GAAGGCAGCCATCTCTGGTGTTCGACAG	
	PSIG1 RT-Primer set B RV	CTGGTAAAGACAACCCACCAGAAC	
	PSIG1 RT-Primer set C FW	GGGGTCCAGTTGAGCAATCAACCCAGG	
	PSIG1 RT-Primer set C RV	CACCTGTGTTGTTTCGGGTCCCGACTCCC	
Constructs of *PSIG1*pro-*PSIG1* vector	*Sal*I site-*PSIG1* promoter FW	AACCAATTCAGTCGACGACCAAACCACGCCAACAAACCTCGT	in this study
	PSIG1 promoter RV	AGTTAGCCATGTCGACGGATTTGATCGGTTTCGAAGTTAC	

Constructs of *PSIG1*pro-*PSIG1* vector	*Sal*I site-*PSIG1* promoter FW	AACCAATTCAGTCGACGACCAAACCACGCCAACAAACCTCGT	Rieshs-Kearnan et al., 2012
	PSIG1 promoter RV	AGTTAGCCATGTCGACGGATTTGATCGGTTTCGAAGTTAC	
	*Sal*I site-*PSIG1* ORF FW	gaaccaattcagtcgacATGGCTAACTCTTCCGCTGGCTCCGCCGCAG	
	*Not*I site-*PSIG1* ORF RV	ATCTCGAGTGCGGCCGCTCAGTCCTCAATTGTCTGAATCTCTCCC	
smg7-4 T-DNA genotyping	SAIL1_LB	GCCTTTTCAGAAATGGATAAATAGCCTTGCTTCC	in this study
	Est1b-15	GCTGCTTCTCTTGCTAGTAGCCTA	
	Est1b-16	TGAGTGCCTACGCATGTGTAAACA	
pENTR4m construction	pENTR4 *Nco*I site mutation FW	GGCTCCACCGCGGGAACCAATTCAGTCGACATG	in this study
	pENTR4 *Nco*I site mutation RV	GGTTCCCGCGGTGGAGCCTGCTTTTTTGTACAA	
Constructs of *PSIG1-GFP* vector	*Not*I site-*PSIG1* stop codon mutation RV	ATCTCGAGTGCGGCCGCCAGTCCTCAATTGTCTGAATCTCTCCCA	in this study
Constructs of ECFP-SMG7	*Sal*I site-*SMG7* ORF FW	AACCAATTCAGTCGACATGATGACTTTACAGATGGATAAAACTACTGCTTC	in this study
	*Not*I site-*SMG7* ORF RV	ATCTCGAGTGCGGCCGCGTCACACAAAGTGACGACTCGACC	
Recombinant protein expression	*Sal*I site-*PSIG1* ORF FW for pGEX-4T-3	GAATTCCCGGGTCGACATGGCTAACTCTTCCGCTGGCTCCGCCGCAGACCAC	in this study
	*Not*I site-*PSIG1* ORF RV for pGEX-4T-3	AGTCACGATGCGGCCGCTCAGTCCTCAATTGTCTGAATCTCTCCC	
	*Not*I site-*PSIG1* 1-606 RV for pGEX-4T-3	AGTCACGATGCGGCCGCTCACGACTTAGCCCGTAAATGTG	
	PISG1 Y575A mutation FW	GCTGGGGCTTTTGGCATAGATTTGCTA	
	PISG1 Y575A mutation RV	GCCAAAGCCCCAGCCTCAAACCATCC	
	PISG1 W570A/Y575A mutation FW	ATTGGAGCGTTTGAGGCTGGGGCTTTT	
	PISG1 W570A/Y575A mutation RV	CTCAAACGCTCCAATGATATCAGATCC	
	*Not*I site-SMG7-N RV	ATCTCGAGTGCGGCCGCGTCAGGGACCTTCAGTTCCAAACG	
	*Sal*I site-SMG7-C FW	AACCAATTCAGTCGACATGAAGGAAAAGAAAGCTCGTATAAAGAG	

Reference

1) Gloggitzer *et al*. Nonsense-mediated mRNA decay modulates immune receptor levels to regulate plant antibacterial defense. Cell Host Microbe 16, 376-390 (2014).

2) Trujillo, M., Ichimura, K., Casais, C. & Shirasu, K. Negative regulation of PAMP-triggered immunity by an E3 ubiquitin ligase triplet in Arabidopsis. Curr. Biol. 18, 1396-1401 (2008).

3) Riehs-Kearnan, Gloggnitzer, J., Dekrout, B., Jonak, C. and Riha, K. Aberrant growth and lethality of Arabidopsis deficient in nonsense-mediated RNA decay factors is caused by autoimmunne-like response. Nucleic Acid Resarch 40, 5615-5624 (2012).
