# Supplementary material for: The GYF domain protein PSIG1 dampens the induction of cell death during plant-pathogen interactions
Source: PLoS Genet. 2017 Oct 26;13(10):e1007037. doi: 10.1371/journal.pgen.1007037 (PMC5657617; doi:10.1371/journal.pgen.1007037)
Supplement: S1 Information — (PDF) [file pgen.1007037.s024.pdf]

Supplementary information 1

Statistical analysis of quantitative PCR data using R

```
setwd("C:/Users/Username/Documents/folder_name/")
getwd()
library(lme4)

input.file <- file.choose()
dat <- read.delim(input.file, header=T)
genotype <- levels(dat$genotype)
treatment <- levels(dat$treatment)
dat_lmer <- summary( lmer( Ct ~ genotype:treatment - 1 + (1|replicate), data=dat))
par(mfrow=c(1,2))
plot(dat[,"Ct"], dat_lmer$residuals)
abline(0,0,col="red")
qqnorm(dat_lmer$residuals)
qqline(dat_lmer$residuals, col="red")
estim <- dat_lmer$coefficients[,1]
write.table(dat_lmer$coefficients, "estim.txt", sep="¥t", row.names=T,col.names=T, quote=F)
#SE of fixed effects = sqrt(diag(vcov(dat_lmer)))
df <- length(dat_lmer$residuals) - length(estim) -    ((as.vector(dat_lmer$ngrps)) - 1 )
vcov <- as.matrix(dat_lmer$vcov) #Calculate Variance-Covariance Matrix for a Fitted Model Object
genotype.treatment <- names(estim)
genotype.treatmentcomp <- c()
for (i in 1:(length(genotype.treatment)-1)){
        for (j in (i+1):length(genotype.treatment)){
                genotype.treatmentcomp <- c(genotype.treatmentcomp, paste(genotype.treatment[i],
genotype.treatment[j], sep=":"))
        }
}
id.mat <- matrix(0, ncol=1, nrow = length(genotype.treatment) )
p.val <- c()
for ( i in 1:(length(estim)-1)) {
  for (j in (i+1):length(estim)){
   id.mat.x <- id.mat
   id.mat.x[ i, 1 ] <- 1
```

```
    id.mat.x[ j, 1 ] <- -1
    stder <- sqrt( t(id.mat.x) %*% vcov %*% id.mat.x )
    t.val <- abs( estim[i]-estim[j]) / stder
    p.val <- c( p.val, 2 * pt( t.val, df, lower.tail=F ) )
    }
}
names(p.val) <- genotype.treatmentcomp
write.table(p.val, "p.val_all.txt", sep="¥t", row.names=T,col.names=F, quote=F)

dev.off()
```
