# Supplementary material for: The GYF domain protein PSIG1 dampens the induction of cell death during plant-pathogen interactions
Source: PLoS Genet. 2017 Oct 26;13(10):e1007037. doi: 10.1371/journal.pgen.1007037 (PMC5657617; doi:10.1371/journal.pgen.1007037)
Supplement: S2 Information — (PDF) [file pgen.1007037.s025.pdf]

Supplementary information 2

Tukey HSD test and visualization of box plot using ggplot2

```
setwd("C:/Users/Username/Documents/folder_name/")
getwd()
library(ggplot2)

# input Trypan blue staining area data
x <- read.csv("C:/Users/Username/Documents/folder_name/file_name.csv", row.names=1)
x
str(x)

by(x$result, x$genotype, summary)
boxplot(result~genotype, data=x)

TukeyHSD(aov(result~genotype, data=x), "genotype", ordered = TRUE, conf.level=0.95 ) # If you would like to calculate p < 0.01, you change the number of conf.level=0.95 to 0.99.
levels(x$genotype)
g <- ggplot(x, aes (x = genotype, y = result))
g <- g + scale_x_discrete(limits=c("Sample_A", "Sample_B", "Sample_C", "Sample_D"))
g <- g + stat_boxplot(geom = "errorbar", width = 0.5, size = 0.5)
g <- g + geom_boxplot(size = 1.0)
g <- g + ggtitle ("Trypan blue staining area")
g <- g + xlab("Treatment")
g <- g + ylab("um2")
g <- g + theme_bw()
g <- g + theme(panel.border = element_rect(fill = NA, colour = "black", size = 1.0))
g <- g + theme(axis.text=element_text(size=5),
               axis.title=element_text(size=14,face="bold"))
plot(g)
dev.off()
```
